# An orchestra of machine learning methods reveals landmarks in single-cell data exemplified with aging fibroblasts

**DOI:** 10.1371/journal.pone.0302045

**Published:** 2024-04-17

**Authors:** Lauritz Rasbach, Aylin Caliskan, Fatemeh Saderi, Thomas Dandekar, Tim Breitenbach

**Affiliations:** Department of Bioinformatics, Biocenter, University of Würzburg, Würzburg, Germany; Ardakan University, ISLAMIC REPUBLIC OF IRAN

## Abstract

In this work, a Python framework for characteristic feature extraction is developed and applied to gene expression data of human fibroblasts. Unlabeled feature selection objectively determines groups and minimal gene sets separating groups. ML explainability methods transform the features correlating with phenotypic differences into causal reasoning, supported by further pipeline and visualization tools, allowing user knowledge to boost causal reasoning. The purpose of the framework is to identify characteristic features that are causally related to phenotypic differences of single cells. The pipeline consists of several data science methods enriched with purposeful visualization of the intermediate results in order to check them systematically and infuse the domain knowledge about the investigated process. A specific focus is to extract a small but meaningful set of genes to facilitate causal reasoning for the phenotypic differences. One application could be drug target identification. For this purpose, the framework follows different steps: feature reduction (PFA), low dimensional embedding (UMAP), clustering ((H)DBSCAN), feature correlation (chi-square, mutual information), ML validation and explainability (SHAP, tree explainer). The pipeline is validated by identifying and correctly separating signature genes associated with aging in fibroblasts from single-cell gene expression measurements: PLK3, polo-like protein kinase 3; CCDC88A, Coiled-Coil Domain Containing 88A; STAT3, signal transducer and activator of transcription-3; ZNF7, Zinc Finger Protein 7; SLC24A2, solute carrier family 24 member 2 and lncRNA RP11-372K14.2. The code for the preprocessing step can be found in the GitHub repository https://github.com/AC-PHD/NoLabelPFA, along with the characteristic feature extraction https://github.com/LauritzR/characteristic-feature-extraction.

## Introduction

Single-cell measurement is a powerful technology since it provides the plurality of gene expression based on cell level. This plurality enables us to get an insight into the variation of expression within tissue and to apply statistical methods. Using these methods makes it possible to distinguish between tissue(phenotypic)-inherent expression variation and variation causing significantly different cell phenotypes. However, this detailed measurement naturally generates a massive amount of data. Due to the number of samples and the potential big size of each sample, it is challenging to identify the significant causes differentiating the phenotypes without augmenting the analysis with suitable mathematical analysis tools to process the data and automate as much of the investigations as possible. For applying such mathematical tools, including machine learning (ML) techniques, which are useful for analyzing big data, the measurements need to be modeled by a corresponding framework.

In such a framework, we can model each single cell as a data point in a multi-dimensional vector space where the dimension equals the number of measured genes/features. Depending on the measure for the expression of each gene in a cell (e.g., transcription (RNA counts) or translation (proteins)), the corresponding data point can be located accordingly. Visualization of the data is one helpful approach to make the encoded measurement information in the high-dimensional vector space accessible to human researchers. For this purpose, several tools have been developed to embed the data points from the high-dimensional space into a plane according to similarity in the expression pattern [1, 2]. The rationale is that cells with a similar expression pattern (or generally similar feature values) should show similar phenotypic behavior and vice versa. In other words, the phenotypically different cells should have a bigger difference in their expression pattern (or in general feature values) than cells from the same phenotype. In the mathematical model, cells with only minor changes in the expression pattern should be located close to each other, while the distance between cells from other phenotypes is supposed to be big. Consequently, with a suitable projection from the high-dimensional vector space into the plane, clear clusters in the plane might indicate distinct different expression patterns that are assumed to map to phenotypes. Examples could be different tissue differentiated from stem cells, heterogeneous tumor tissue with corresponding tumor subtypes, or different immune cells.

Once these clusters are defined, the next question is this: What are the significant differences in the feature space (single-cell expression data) that made the clustering algorithms generate their results? The corresponding explanation might reveal the causes for the cells developing different phenotypes. To find features (expression of genes) that carry the significant information for cluster differences, various feature selection methods have been developed. Please see the introduction of Caliskan et al. (2023) [3] for an exemplary compilation. However, all such methods identify features correlating with the difference between the clusters.

Extending previous work [3], we here introduce a new data analysis concept to really understand which minimal gene sets objectively (meaning with a defined mathematical framework instead of a human manually selecting them via, e.g., intuition from all differentially expressed genes) separate which different clusters and why this is the case (causal reasoning). To achieve this, (i) unlabeled feature selection objectively determines groups, including minimal gene sets separating different groups. (ii) Next, ML explainability methods facilitate transforming the features correlating with phenotypic differences into causal reasoning since these tools make the feature values that made the ML decide to assign a cell a phenotype more transparent. This insight into the expression values can be used as a starting point for causal reasoning by human researchers. (iii) This is further supported by additional pipeline and visualization tools allowing user knowledge to be integrated, which further boosts causal reasoning and explainability.

In order to find options to influence a cell’s behavior in a desired manner, like a therapy, it might be one helpful approach to identify the causality for the difference since this may provide options to turn one phenotype into another. Small and meaningful results provided by such methods support researchers in finding these causes, thus enabling an explanation of the mode of action. A plurality of methods for feature selection, each working with different mathematical selection mechanisms relying on different assumptions, provides different meaningful and small gene selections. If finding an explanation for the separated phenotypes from one gene selection is challenging, different alternatives might be useful.

This procedure can be enriched with techniques from ML explainability, e.g., SHAP [4] or LIME [5]. The concept of explainability helping to identify causal relations works as follows in our framework: If we train a model based on labels associated with the clusters and the selected genes and the model can classify the cells with a high accuracy based on the selected genes, then the corresponding genes provide the relevant information about the phenotypic differences. However, the rules of how exactly gene expression values allow for a classification are hidden in the model. We can support the extraction of such rules by facilitating the analysis of causalities for the different cell behaviors with the ML explainability since these methods aim to make the learned rules more transparent. These methods visualize which the most important gene expression values for a decision are, i.e., based on the expression profile of the cell, what made the model classify a cell as it did. Consequently, such techniques facilitate the analysis of gene expression patterns (like genes that are always highly expressed and others lowly) characteristic of a cluster or phenotype.

Our technical aim is to provide a software pipeline that purposefully combines all these data analytic tools within one modularized platform to visualize results and information for single-cell analysis. These visualizations allow the extraction of significant differences in expression profiles to facilitate finding explanations for phenotypic behavior. The modularization is generated by providing a clear input and output for each method whose implementation we provide in our GitHub repository. Consequently, each stage of our pipeline (1 to 9, defined below) can be filled with suitable methods for the concrete task described in this stage. Furthermore, we would like to showcase its application.

In our showcase, we will analyze single-cell data provided by Solé-Boldo et al. (2020) [6] (the RDS file is available via the GEO database: https://www.ncbi.nlm.nih.gov/geo/query/acc.cgi?acc=GSE130973), who analyzed human skin fibroblasts from a sun-protected area of healthy ‘young’ donors (25 and 27 years old) and healthy ‘old’ donors (53, 69, and 70 years old) using single-cell RNA sequencing and found age-related changes in fibroblast subpopulations [6], using the conditions ‘young’ and ‘old’.

Comparing these two conditions allows the discovery of changes in gene expression that might be caused by chronological aging. This is especially interesting, as Garmany et al. (2021) recently reported a healthspan-lifespan gap of about 9.2 years [7]. Thus, individuals will live about one-fifth of their lifetime suffering from morbidities [7]. Furthermore, 79% of the overall years are lived with disability, and 71% of the worldwide annual deaths are due to chronic diseases. The majority of chronic disease-related deaths (80%) can be attributed to four common conditions: diabetes, cardiovascular diseases, chronic respiratory diseases, and cancer [7], all of which are associated with aging [8].

With the predicted global increase of ‘aging’ (countries with more than 10% of the population being 70 years of age or older) or even ‘advanced aging’ (more than 20% of the population ≥70 years of age) [7], understanding aging and age-related changes, and subsequently finding possible treatments or even cures for aging is of utmost importance. Thus, especially intrinsic aging, which is not due to external factors, is of great research interest.

Solé-Boldo et al. (2020) obtained all skin fibroblasts from the typically sun-protected inguinoiliac region of male Caucasian donors [6]. Since the authors intended to focus on intrinsic aging, they ensured that none of the donors had received UV therapy or showed any signs of sun exposure, such as tanned skin or actinic skin damage [6]. Additionally, the medical records of the donors were carefully reviewed, focusing on any skin diseases or conditions that could affect the skin, and all donors underwent a full body skin examination by a dermatologist [6]. None of the donors had a history or showed signs of systemic or inflammatory skin disease [6]. Therefore, changes in gene expression between the ‘young’ and the ‘old’ samples are most likely due to chronologic, intrinsic aging and not caused by external factors, including UV exposure, which is the most important external cause of skin aging (extrinsic aging) [7, 8].

Different fibroblast subtypes, such as papillary fibroblasts (located in the superficial papillary dermis) and reticular fibroblasts (located in the reticular dermis), not only differ in their morphological characteristics but also exhibit distinct characteristics, including proliferation rates, their abilities to produce and respond to growth factors and cytokines, and their expression of extracellular matrix (ECM) components [6]. In their skin samples, Solé-Boldo et al. (2020) identified several cell clusters, including four fibroblast clusters (#1, #2, #3, and #9 in their publication, identified by the archetypal markers DCN, COL1A2, LUM, PDGFRA, and VIM) containing distinct fibroblast subtypes with distinct functional roles [6]. The four fibroblast subpopulations showed differential mesenchymal, secretory, and pro-inflammatory annotations, which became reduced with age [6].

In mice, Salzer et al. (2018) observed age-related changes in fibroblast subpopulations [6, 9], which also appeared to be influenced by the systemic metabolism [9]. Upon aging, the two fibroblast subpopulations became less well-defined and gained adipogenic traits while their expression of ECM genes was reduced [6, 9]. In human skin samples, fibroblast subpopulations had already been identified in a chronically-sun-exposed skin region (dorsal forearm) in a heterogeneous group of donors [6, 10] and in the abdominal skin of a single female donor [6, 11].

Besides an age-related loss of cell identities, Solé-Boldo et al. (2020) also observed the expression of skin aging-associated secreted proteins (SAASP) in old fibroblast subpopulations [6]. As the specific age-related changes in fibroblasts were well-established, they focused on the expression profiles of young fibroblasts, which showed strongly enriched classical fibroblast functions related to the extracellular matrix (ECM) and collagen production [6].

The aging process of fibroblasts and the hallmarks of fibroblast aging have been thoroughly categorized by Tigges et al. (2014) and include ECM-remodeling, which can be observed in fibroblasts in culture and *in situ*, EGF insensitivity, increased protein secretion (SASP), and cellular senescence [12]. Fragmentation of the dermal ECM and a decrease in collagen production are also associated with skin aging [13]. Additionally, fibroblast dysfunction and the corresponding dermal ECM remodeling have been linked to age-related skin changes, such as the formation of wrinkles [12].

For our example, we chose the fibroblast clusters of the single cell sequencing data published and shared by Solé-Boldo et al. (2020) [6] for several reasons: Fibroblasts undergo age-related changes and all four fibroblast clusters contain ‘young’ and ‘old’ cells, thus, differences between ‘young’ and ‘old’ cells are most likely due to aging and not due to differences between separate clusters. Since our ML method does not require the cells to be labeled according to their condition or cell type, we use this example to check and demonstrate whether our algorithm is able to distinguish between ‘young’ and ‘old’ cells solely based on the sequencing data without additional information such as labels. Additionally, we analyze the genes that were identified as particularly relevant to the differences between the two conditions, focusing on their roles in aging and fibroblast aging.

**Fig 1:**
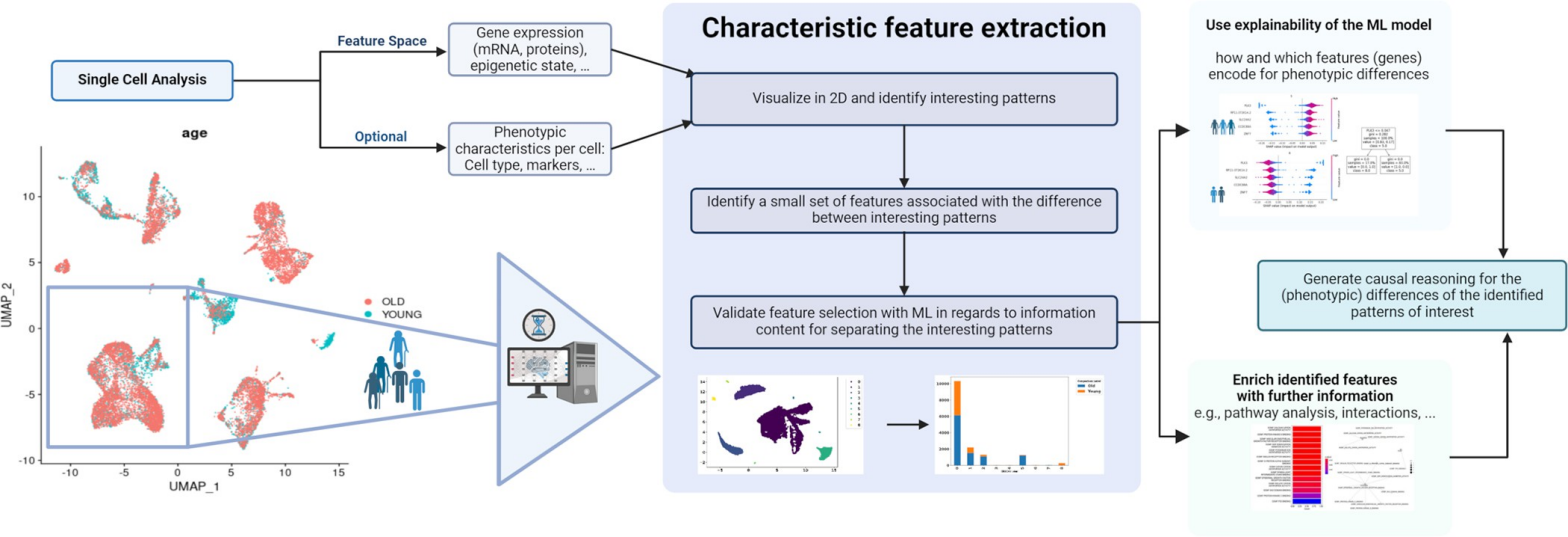
Visualization of the analysis workflow.

With our ML method, we intend to contribute to the growing toolset of gene analysis methods by introducing a user-friendly approach that has the potential to uncover novel genes of interest. As indicated by our previous proof of principle study, the PFA and mutual information have demonstrated their capability to identify potentially interesting genes that might not receive high rankings using other conventional techniques [3]. Therefore, we intended to enhance the usability, potentially allowing “a quick first glance” at potentially interesting additional genes. Moreover, we also tackle the far more challenging task of identifying the best features without previously labeling groups, so the pipeline should also deliver this step. Finally, as we use ML methods, we have a refined toolbox to deliver an explainability of our analysis–a useful addition that is also integrated into our pipeline. The general workflow is visualized in Fig 1.

In this work, we present a single-cell analysis pipeline consisting of multiple parts that can be extended in each stage with further methods:

1. **Feature reduction:** The intention is to reduce the dimensionality of the input feature space to genes that carry the information. Our implantation uses the principal feature analysis (PFA) [14] because of its interpretability since only genes that depend on the expression of other genes and can therefore be seen as functions of other genes are removed. Thus, the information they carry can be constructed by the other genes. There are other dimension reduction techniques like autoencoder [15] or principal component analysis (PCA) [16] that can be used at this stage. However, these methods provide a transformation of the original genes, which is challenging for interpretation since one needs to take the fitted transformation into account. There is evidence that clustering techniques, like UMAP [1] (‘curse of dimensionality’) or MARS [15] (see the end of “Overview of MARS” in the Methods section), benefit from feature reduction. However, performing a feature reduction before the UMAP embedding is not strictly necessary. With our implementation, we can easily try its effects on the embedding and evaluate if it benefits from feature reduction.
2. **Embedding:** The intention of this stage is to provide an embedding of the high dimensional input vector space, where each gene (or, in general, each measured feature) of the single cells is represented by a dimension, into a plane or three-dimensional space for the purpose of visualizing similar expression patterns. An important property of the embedding is that similar expression profiles are close in the low dimensional space. Thus, corresponding single cells are clustered together if their expression is similar and far away if it is not similar. In our implementation, we use the UMAP method for this purpose. Such a low-dimensional space can be visualized, and thus, the user can have a first impression of whether the data contains interesting information or not.
3. **Labeling:** With a density-based clustering algorithm, we can automatically associate each cluster with a label. These labels will allow us to analyze characteristic genes that separate the clusters/labels. In our implementation, we use DBSCAN [17] or HDBSCAN [18]. The rationale is that a cluster is defined by a surrounding space where there are no data points and consequently the density of data points differs significantly between clusters. A selection by coordinates is also possible, e.g., all data points within a box can be associated with the same label. Furthermore, any other method that annotates/names/labels single cells can be used here, e.g., Brbic et al. (2020) [15] (see “Overview of MARS”).
4. **Cluster comparison with further labels:** This stage is intended to perform either a sanity check for known phenotypes or to identify interesting clusters in the case where nothing about subtypes is known, like a heterogeneous tumor tissue. For example, we can sanity check that the found clusters match known phenotypes or other phenotypic properties that coincide each with high purity in separate clusters, meaning that the features (e.g., gene expression) provide powerful information to separate the phenotypes. If not, the current feature space (e.g., gene expression) might not contain relevant information regarding the phenotypic differences of interest and could be extended purposefully or the embedding might cluster according to other properties that are not known. In another example with tumor tissue, we can check if some clusters identified from the pipeline so far have a high purity of additional labels, like adult or pediatric. Then, these clusters can be selected for further analysis to get the expressional differences which might lead to better therapies by finding and targeting more relevant genes. This stage is optional and is not necessary for the rest of the pipeline, in particular, if no further properties than gene expression is available. However, it may be helpful in some cases, like investigating only differences between interesting clusters or fine structures within one cluster (see Discussion for details).
5. **Data splitting:** Once interesting clusters are identified for the causal analysis of expressional differences, we would like to ensure that the analysis is robust with respect to data sampling. For this purpose, we have the option at this stage to split the data into subsamples, use one for the analysis, and hold the others back for validation and explanation to check that results based on the selected features/genes are independent of the used subsample and thus generic. This step is optional and requires a sufficiently large number of cells in the investigated clusters such that the statistical properties of the subsamples do not change too much due to a small sample size.
6. **Feature selection related to labels/output function:** After labels have been created and selected for analysis, we further select genes that are not independent of the label and sort out all others for further analysis. We use a chi-square test for this purpose.
  In order to lower the number of selected genes in this step, removing redundancy in stage 1), e.g., with the PFA, might be beneficial. In case methods like PCA or autoencoder have been used before the embedding stage, applying PFA to the gene expression data set prior to gene selection by the chi-square test in this stage might help reduce redundancy and produce a smaller model of the phenotypic difference-generating genes. Consequently, we could apply the workflow as presented in Caliskan et al. (2023) [3], where labels are given a priori. However, the analysis could be performed with other methods of redundancy reduction or even without it.
7. **Mutual information:** In order to further reduce the feature/gene selection, mutual information has been shown to be a useful concept for ranking genes whose expression shares information about the labels/output function [3]. Mutual information asks: How much information does one gain about the label given the expression value of the corresponding gene? By taking only the best-ranked genes, we can balance model accuracy and model size based on the selected genes.
8. **Validation:** In order to check that all our stages have not deleted important information while selecting features/genes, e.g., by having set the threshold for the mutual information too high or due to failure of some feature selection, we validate whether a model can learn to separate clusters/predict the output function value based on the selected genes. If the accuracy of the trained model is sufficiently high, we know our selection contains enough information, which means that the corresponding relations between gene expression and label (phenotypic difference) can be constructed from the selected information. In case we have used data splitting, we can repeat just the validation step with the selected features on other sets and check for comparable accuracy. If yes, we can say that our selection is robust with respect to the sampling. In our Python implementation, we use standard Keras ML models like MLP. If the accuracy is high enough, we can argue that we have all the necessary information in our gene model. However, the opposite is not correct since model training could fail due to, e.g., a bad hyperparameter selection. Furthermore, any procedure for gene selection can be validated with that procedure. In our case, we use the ML framework from Python as a well-developed framework for function approximation, which is the relation between the genes and the corresponding output function, e.g., the label function that assigns each single cell a label. Explanation of this relation is of high interest since it might encode the causal relations for the difference.
9. **Explainability:** In order to analyze the rules encoded in an ML model that allow us to conclude the corresponding output function value, like the cluster assignment, from the expression of the genes, we implement a model explainer like the SHAP framework or tree explainer (only for decision tree). These techniques enable us to analyze if, e.g., some genes are always highly expressed and others lowly if the model decides on a corresponding label. These methods provide information on what is important for the model decision. Since the model is only based on correlation and thus not necessarily a causal model, different implemented methods may provide different approaches for explaining the causalities in case the results of one method are challenging to interpret. If we used the data splitting before, we can check whether we come to the same explanation/feature importance pattern independent of the used subsample data set. A chain of explanation is not limited to the genes extracted in the pipeline but can also serve as a starting point in a complex scenario to construct a meaningful reasoning, including methods such as pathway analysis.

In summary, we combine (i) unlabeled feature selection and minimal gene sets, separating different groups with (ii) ML and explainability methods and (iii) further pipeline and visualization tools, allowing user knowledge to be iteratively integrated.

Hence, we deliver a powerful framework that combines unsupervised learning, like UMAP or autoencoder and feature selection, with supervised learning to validate findings and apply tools from explainability to help us identify correlating genes and facilitate causal analysis of phenotypic differences.

Furthermore, we bring well-established methods, like UMAP, together with new purposeful extensions to analyze their results more deeply, e.g., getting differences between the UMAP clusters with the chi-square test and mutual information in a modularized software platform for easy application and further development.

Our work does not aim at cell annotation but provides a framework for analyzing the gene expression differences between phenotypes given by a small and meaningful set of genes. We assume that a phenotype is characterized by a similar expression pattern of the corresponding single cells. However, our method is generic and can be used to provide a small feature set that carries information about differences. Consequently, the set of gene expressions can also be enriched by other features like methylation states or other epigenetic properties to enrich the data model of a single cell apart from gene expression data.

We focus on visualization to identify interesting (sub)clusters, specifically highlighting the characteristic genes.

Our implementation, provided with that work, is a minimal working implementation to focus on the concept. Since we provide the code via a GitHub repository (links are given in the Abstract), adaptions depending on use cases can be done easily, and the implementation can be purposefully developed, like including further feature selections. The clearly defined inputs and outputs facilitate the extension.

The application of our implementation using the skin fibroblast data set generated by Solé-Boldo et al. (2020) [6] provided a selection of genes that might be of interest for further research. Since they appear to be involved in aging-related changes in gene expression and were identified by our method as being most relevant for discerning the differences between both conditions (‘young’ and ‘old’), they might play important roles in the aging process.

Our method is generic and can be used to provide a small feature set that carries information about differences. It is particularly useful in single-cell sequencing and other high-dimensional data sets, including phenotypic information. It is unique because it provides visualization, checkpoints, and possibilities to use biological domain knowledge to recheck and improve the clustering at each pipeline step. This will open numerous applications and booster omics analyses, especially when fine structures of clusters are present.

## Methods

Our pipeline consists of several methods, which are described below. In a nutshell, this is a pipeline consisting of supervised and unsupervised machine learning. Moreover, it includes stochastic methods for the purpose of single-cell analysis. Our main goal is to cluster single cells according to their expression profile and to provide a small but meaningful set of genes that contain the relevant expressional differences between these clusters. The rationale behind our approach is that phenotypically similar cells have a similar expression profile. Since the behavior of each single cell within a phenotype is similar, our rigorous definition of single cells according to their expression profile and expressional difference might provide an additional approach to finding treatments and drug targets to influence each phenotype in a desired manner.

In the first step of our pipeline, we remove genes with a constant expression level across all cells (since they provide no information because they are expressed the same for any phenotype in our data), and we remove redundancy. We use Algorithm 2 from the Principal Feature Analysis (PFA) [14]. The main idea of the PFA is to consider expressions of genes as functions, which have other genes as arguments. For example, we have a gene that is regulated by other genes. Then, the expression level of the regulated gene can be seen as a function of the other genes’ expression levels. Once we know these values and the dynamic according to which the regulation takes place, we can provide the expression value as a function of the input/argument genes. With the PFA, we can detect genes whose expression is just a function of other genes. As those genes do not provide any further information, we are able to remove them, which reduces the dimensions and complexity of the single-cell data. By retaining only genes whose expression levels are independent of each other, we ensure that the information provided about the differences between the clusters/phenotypes is non-overlapping and free of redundancy. The main ingredients for the PFA are the following.

### Discretization of expression ranges

An important step is to discretize the continuous gene expressions (binning) [14]. This step is required to use the chi-square test for independence and mutual information later on because the continuous expression levels of the genes need to be discretized due to the fact that the chi-square test and mutual information can only be applied to discretized data or investigate the correlation of events, respectively. For this purpose, we implement Algorithm 4 from Breitenbach et al. (2022) [14] in our pipeline. The essential parameter of this algorithm is min_n_datapoints_a_bin, defining the minimum number of data points, where each data point is the measurement of the considered gene within one single cell that is assigned to a bin during this process. Each bin is a discretized ordered category modeling the strength of the gene expression of the corresponding gene. For every gene expression, we determine the minimum value *m* and maximum value *M* over all single-cells per gene (or in general feature). If *M*≤*m*, the expression of the corresponding gene is constant and this gene can be removed from the list of genes later used for an explanation of phenotypic differences as the data of the gene without any variation does not contain information that could explain variation in a label of single cells. If *M*>*m*, we iterate over the data points in ascending order and assign at least the minimum number of data points (min_n_datapoints_a_bin) to a bin. If one bin has at least min_n_datapoints_a_bin number of data points and the next value in the ascending order is equal to the current value, it is also assigned to the current bin until the following value is larger than the current one. If there are fewer values left than min_n_datapoints_a_bin, they are all put into the last bin opened. The selection of min_n_datapoints_a_bin is important for the computation time and the transferring of information from the continuous expression values to discrete expression levels/events. If the parameter is too large, this can result in a small number of bins, and the structural properties of the original continuous data get lost, meaning a too rough discretization of the continuous expression level into discrete expression levels of the corresponding gene. If the parameter is too small, there is a risk of creating too many bins with too few expected data points in an entry of the corresponding contingency table, see the explanation for the chi-square test below, violating needed assumptions. More specifically, this can result in dropping below the recommended threshold of five expected data points in a joint bin [19] for the chi-square test of independence. Illustratively, the expected frequencies of the corresponding contingency table with the joint events (e.g., gene A has expression x and gene B has expression y) could fall below the threshold because the data points of a bin of one gene (modeling a certain expression range) are split among the other bins of the other gene to define the joint expected frequencies.

### The chi-square test of independence

The mathematical model for the chi-square test of independence of two gene expressions looks as follows.

We assume that we measure the expression of each gene in *N*∈ℕ single cells. The expression value of each gene is modeled by the random variable *X*_*i*_: Ω→*Z*_*i*_, *i*∈{1,…,*n*}, *n*∈ℕ number of genes. Illustratively, this random variable *X*_*i*_ takes the value of the corresponding gene *i* whenever this gene is measured in a single cell (or, in general, any feature of the cell). Consequently, each measurement of expression values of genes in a single cell is a random experiment in which the corresponding random variables modeling the corresponding genes take the corresponding expression values. Due to the discretization of the expression level of each gene, *Z*_*i*_ is a set of bins $z_{k}^{i},\;k\in \{1,\ldots ,m_{i}\},\;m_{i}\in N$ number of bins in which the range of expression values is discretized for gene *i*. Illustratively, the space *Z*_*i*_ is the space of possible discrete expression values of gene *i* based on the selected discretization (see “Discretization of expression ranges”). Furthermore, each $z_{k}^{i}$ models a concrete range in which expression levels of gene *i* are assigned to the discrete expression level *k*. Now, fix a gene *i* and a gene *j*, *j*∈{1,…,*n*}. Within *N* single cells, let *O*_*kl*_∈ℕ be the observed frequency that the expression level of gene *i* is in the bin $z_{k}^{i}$ and that the expression level of gene *j* is in the bin $z_{l}^{j},\;l\in \{1,\ldots ,m_{j}\}$ with $N=\sum_{k=1}^{m_{i}}\sum_{l=1}^{m_{j}}O_{kl}$. By dividing the observed frequency by the number of all measurements *N* (number of measured single cells), the corresponding probability that when measuring a single cell, gene *i* of that cell is expressed on level $z_{k}^{i}$ and gene *j* of that cell is expressed on level $z_{l}^{j}$ is defined by

$$P(X_{i}=z_{k}^{i}\wedge X_{j}=z_{l}^{j})≔\frac{O_{kl}}{N}.$$

Furthermore, let $P(X_{i}=z_{k}^{i})$ be the probability that the expression level of gene *i* is within the bin $z_{k}^{i}$, which is calculated by dividing the number of all single cells where the expression value of gene *i* is within the bin $z_{k}^{i}$ by *N*,

$$P(X_{i}=z_{k}^{i})≔\frac{1}{N}\sum_{l=1}^{m_{j}}O_{kl}.$$

An analog definition for

$$P(X_{j}=z_{l}^{j})≔\frac{1}{N}\sum_{k=1}^{m_{i}}O_{kl}.$$

Under the assumption that the expression of gene *i* is independent of gene *j*, the expected frequency *E*_*kl*_∈ℝ that the expression level of gene *i* is in the bin $z_{k}^{i}$ and that the expression level of gene *j* is in the bin $z_{l}^{j}$ is given by

$$E_{kl}≔P(X_{i}=z_{k}^{i})P(X_{j}=z_{l}^{j})N.$$

The rationale is that, under the assumption of independence, from *N* single cells, gene *j* is expressed in $z_{l}^{j}$ in the fraction $P(X_{j}=z_{l}^{j})$, and from this number, given by $P(X_{j}=z_{l}^{j})N$, the fraction of $P(X_{i}=z_{k}^{i})$ single cells have expressed gene *i* in $z_{k}^{i}$. However, this calculation holds only true if the expression of gene *j* does not influence the expression of gene *i* where in this case the fraction $P(X_{i}=z_{k}^{i})$ does not depend on *j*.

If the expression of the genes is independent of each other, observed and expected frequencies are supposed to be equal for all *k*∈{1,…,*m*_*i*_} und *l*∈{1,…,*m*_*j*_} or equivalently,

$$P(X_{i}=z_{k}^{i}\wedge X_{j}=z_{l}^{j})=P(X_{i}=z_{k}^{i})P(X_{j}=z_{l}^{j})$$

for all *k*∈{1,…,*m*_*i*_} and *l*∈{1,…,*m*_*j*_} is supposed to hold. Due to noise from the measurement and interplaying dynamics, these equalities might not be fulfilled exactly, even if the assumption of independence is true. Assuming the expression of the genes is independent, variations of (*O*_*kl*_−*E*_*kl*_) around 0 are random due to noise or alternatively (*O*_*kl*_−*E*_*kl*_)^2^>0 even in the case of independent expression.

Consequently, a statistical test is required to decide for independence based on a level of significance, e.g., the chi-square test with the test statistic,

$$\chi^{2}=\sum_{k=1}^{m_{i}}\sum_{l=1}^{m_{j}}\frac{{\left(O_{kl}-E_{kl}\right)}^{2}}{E_{kl}}$$

to decide if the deviation from observed and expected frequencies is likely to come from the noise or rather is caused by the fact that the expression is not independent.

If *E*_*kl*_≥5 for all *k*∈{1,…,*m*_*i*_} and *l*∈{1,…,*m*_*j*_}, then $\frac{O_{kl}-E_{kl}}{\sqrt{E_{kl}}}$ is sufficiently normally distributed, please see, e.g., the Appendix of Breitenbach et al. (2022) [14], and thus the test statistic *χ*^2^ is sufficiently well distributed according to a chi-square distribution with (*m*_*i*_−1)(*m*_*j*_−1) degrees of freedom.

If *χ*^2^ is too big related to the added free varying terms and thus it is too unlikely (based on an a priori fixed p-value) that variations come only from noise, we reject the hypothesis that the expression of the two genes *i* and *j* are independent and assume that the expression of genes is not independent.

### Principal feature analysis

For the principal feature analysis, a dependence graph is constructed by Algorithm 2 described in Breitenbach et al. (2022) [14], following the discretization procedure. The generation of the graph works as follows. Each gene (in general feature) is assigned a node. The edges of the dependence graph are constructed by applying the chi-square test of independence to all pairs of genes, each based on the discretized expression range. The result of a chi-square test of independence on two genes is then used to evaluate the independence of the two gene expressions. If the p-value of the chi-square test for independence–under the hypothesis that the genes are independent–lies above a parameter α (in the current implementation 0.01), the gene expressions are considered independent. In the other case, the hypothesis that they are independent is rejected because a small p-value indicates that given the data and under the hypothesis of independent gene expression getting the observed chi-square value is too unlikely, and we should rather assume the opposite. In this case, the nodes that model the corresponding genes in the dependence graph are connected by an edge. If the hypothesis of independence is not rejected, the corresponding nodes are not connected by an edge.

This graph is then dissected using a minimal cut algorithm until only independent nodes are left; see Breitenbach et al. (2022) [14] for details.

Due to the polynomial complexity of the minimal cut algorithm, we only treat a subset of the dependence graph at a time. A parameter cluster_size allows us to define the maximum size a dependence subgraph can have. Selecting cluster_size too large may result in longer computation time as subgraphs are big. Selecting cluster_size too small may lead to a large remaining dependence graph if no more genes can be removed from the remaining subgraphs, which means more computation time until the entire dependence graph is processed as well.

The principal feature analysis dissects the dependence graph until only complete subgraphs are left (meaning each gene is not independent of each other within such a graph). While the original implementation of the PFA preserves that structure, we adapted the code such that we break the graphs considering each gene being returned by the PFA as a dimension of the vector space where each dimension models the expression level of a gene for the next processing step. The next step is embedding the single cells based on the reduced or original feature space, which requires such a vector representation in our case.

### Embedding with UMAP

After the redundancy is minimized and the remaining genes contain information that is independent of each other, we want to project those genes from a high-dimensional space into a plane. We remark that in this step, no discretization of the expression levels is required. Due to this projection, we are able to visualize the gene expression of the cells. We assume that single cells with a similar expression pattern are phenotypically similar and, as such, show similar behavior as well as cluster accordingly in the plot. Once we are able to group phenotypically similar cells distinctly from other phenotypes into a plane, thus visualizing these groups, we can superimpose the clusters with other phenotypic labels. Thus, we can visually check for interesting patterns where it might be useful to get the difference between these clusters with interesting patterns distilled from the several thousands of genes in a small and meaningful set of genes. These differences could be the start of a causal reasoning for the differences and how to influence cell fates such that we can influence their development into a cluster belonging, meaning expressing different phenotypic properties.

For the purpose of projecting the high-dimensional space in which gene expression is encoded into a plane for visualizing interesting structures, we use the Uniform Manifold Approximation and Projection (UMAP) [1] method. UMAP takes the high-dimensional cell data and optimizes the arrangement of the data points in the plane such that single cells that are close together in the high-dimensional space, meaning having a similar expression pattern, are also close in the plane. Analogously, what is far away in the high-dimensional space is also far away from each other in the plane. Mathematically, this is done by optimizing the following objective, called cross entropy,

$$\sum_{i=1}^{N}\sum_{j=1}^{N}\left[p_{ij}\log\frac{p_{ij}}{q_{ij}}+\left(1-p_{ij}\right)\log\frac{1-p_{ij}}{1-q_{ij}}\right]$$

where *x*_*i*_∈ℝ^*n*^, *i*∈{1,…,*N*} are the data points in a high dimensional space ℝ^*n*^, the measure for being close is given by,

$$p_{i|j}=e^{-\frac{d(x_{i},x_{j})-\rho_{i}}{\sigma_{i}}},$$

*d*(∙,∙) is a metric (e.g., Euclidean distance), *ρ*_*i*_ is the distance to the nearest neighbor and *σ*_*i*_ is set such that

$$\sum_{j=1}^{k}e^{-\frac{d(x_{i},x_{j})-\rho_{i}}{\sigma_{i}}}=\log_{2}\left(k\right)$$

with fixed *k* being the number of nearest neighbors for each data point.

Illustratively spoken, each data point *i* is the measurement of a single cell where the corresponding expression values of the genes are modeled by the vector *x*_*i*_, where each dimension is assigned a gene. The distance measure *d*(*x*_*i*_, *x*_*j*_) is a proxy for the similarity of expression patterns because if the expression of the corresponding genes is similar (e.g., the cells have the same phenotypic behavior), then the distance is small, while in cases of cell *i* and cell *j* have different phenotypic properties, the distance is supposed to be big.

With such a set of parameters, the *p*_*i*|*j*_ ranks the nearest neighbors, canceling out the dependence on the distance between points, making the map “uniform”. Furthermore, we symmetrize

$$p_{ij}=p_{i|j}+p_{j|i}-p_{i|j}p_{j|i}$$

and set

$$q_{ij}=\frac{1}{1+a{\left|\left|y_{i}-y_{j}\right|\right|}_{2}^{2b}}$$

where *y*_*i*_∈ℝ^*d*^, *i*∈{1,…,*N*} are the corresponding vector representations of the data points *x*_*i*_∈ℝ^*n*^, *i*∈{1,…,*N*} in the low-dimensional space ℝ^*d*^ (e.g., plane) and *a*, *b* are chosen with a least square fitting to the curve

$$\left\{ \begin{array}{c} 1,\;if{\left|\left|y_{i}-y_{j}\right|\right|}_{2}<c\\ e^{-{\left|\left|y_{i}-y_{j}\right|\right|}_{2}-c},\;otherwise \end{array}\right.$$

where *c* (min-dist) is the desired separation in the low dimensional space where ||⋅||_2_ represents the Euclidean distance. The above objective is minimized if *q*_*ij*_ (meaning which pair *i*, *j* is close in the low-dimensional vector space) is chosen similarly to the corresponding *p*_*ij*_ (which pair *i*, *j* is close in the high-dimensional vector space), meaning that the topology of the data points (each pair *i*, *j*) of being close or far in the high-dimensional is reflected in the low-dimensional vector space as well.

Alternatively, there is also the t-SNE method [2] implemented in our pipeline, which works similarly to UMAP.

By first removing redundant genes using the PFA, we reduce the dimensionality of the input space. This can be an important step in order to counteract the curse of dimensionality. Reducing the input space reduces the complexity and allows the UMAP method to achieve faster and possibly more accurate results [15, 20].

### Labeling of the clustering

After UMAP leaves us with a two-dimensional representation of the cells, we now aim to automatically assign labels to the cluster, resulting from the UMAP embedding/clustering, with some suitable method to process the clusters subsequently in our pipeline. Clustering allows us to identify and label groups of cells with a high similarity with regard to their feature space (gene expression values). Similarity means that their expression pattern has placed them in a neighborhood with each other in the original feature space (all expression values). Hence, the UMAP method has plotted them together in the plane, at the same time separating them from those not in a neighborhood, i.e., with a different expression pattern. Consequently, there are density variations/drops (data points per area) between the different clusters of single cells, projected by UMAP into a plane, defining the clusters. This is the case because the clusters are separated, meaning that there are areas where there are no cells, resulting in a density of zero. For the next processing step, we use the Density-Based Spatial Clustering of Applications with Noise (DBSCAN) [17] algorithm to identify the clusters separated by density drops while not having to assume spatial properties or characteristics regarding the shape of the clusters.

DBSCAN introduces a criterion using a minimum number of samples (cells) and a radius ε to define a minimum density of cells per area a cluster should have. Clearly, the spaces with no cells around the clusters (density equals 0) are below such a threshold.

The algorithm starts by picking a cell at random. If that cell has the minimum number of cells in its radius, their entirety is considered a cluster. It then repeats this process with each cell in the cluster. Once no more cells are added to the cluster, DBSCAN opens up a new cluster outside of the previous ones. When DBSCAN is finished, each cell is assigned a label. This label either assigns them to a cluster or marks them as noise.

As an alternative, we consider the Hierarchical Density-Based Spatial Clustering of Applications with Noise (HDBSCAN) [18] algorithm. HDBSCAN is very similar to DBSCAN. It utilizes hierarchical clustering in order to become more robust to noise and to density variations within a cluster as well as between clusters since we are not sensitive in this case if a density drops below the criterion of DBSCAN describing the number of cells within a circle with radius *ϵ* potentially causing several labels within one cluster, which could be rather due to noisy variations and thus not desirable or due to a fine structure within a cluster, which can be further analyzed with the subsequent pipeline. HDBSCAN only requires the minimum number of samples (cells) within a cluster for its criterion.

### Identify relevant genes for separating labels

The output of the previous step leaves us with labels for our cell data. We can consider the function that assigns each cell a label/cluster as a function where the expression levels of genes are the arguments. Next, we would like to generate (“learn”) the function that provides the label of a single cell given the expression level of relevant genes. For this purpose, we can select at least two labels for which we would like to get relevant genes from whose expression levels we can assign a single cell to one of the selected clusters. For this purpose, we use a chi-square test of independence between two features where the label is considered as one feature and the second one is the expression level of a gene.

For the discretization, we again use Algorithm 4 from Breitenbach et al. (2022) [14] with the same setting as in the PFA selection stage. This time, the binning is applied to the gene expressions and the label. As the label is already discrete, the algorithm handles this automatically. The considerations for min_n_datapoints_a_bin hold as discussed above, while we recommend the same value as used for the PFA if possible. The chi-square test of independence allows us to check for statistical independence between a gene and the label. When applied to each combination of gene and label, we are able to separate between genes that are statistically independent of the label and those that are not.

As those unrelated genes do not provide any further information, we can safely remove them. This is an important step as it not only reduces the dimensions even further but also leaves us with a set of genes that are relevant to the cluster differences, allowing for a better understanding of the data.

### Mutual information

In the last step, we can calculate the mutual information each remaining gene shares with the labeling. The mutual information models the information about the label knowing the expression value of the gene and vice versa. For a mathematical definition and more explanation, please see Caliskan et al. (2023) [3]. The purpose of the calculation is to balance between the size of the gene set modeling the difference between the labels (phenotypes) and the accuracy of the classification that is possibly based on the selected genes. When ranking the genes according to mutual information that they have in common with the labels and selecting genes above a threshold of mutual information, significant information for the difference is lost at a certain threshold. Consequently, the corresponding function mapping the expression levels of these genes to the labels becomes more and more an approximation the higher the threshold is. The results of the mutual information calculation show us how relevant a gene is to a cluster difference and allow for further reduction of genes with very low mutual information. A small set of meaningful genes improves explainability.

### Validation

In order to validate the results, we chose to append a validation script. Using this script, we would like to evaluate whether the resulting set of genes contains enough information about the difference between the detected clusters of interest. For example, the threshold for mutual information was not too high, and not too many important genes were cut out. The validation uses machine learning on the gene expressions of the selected genes in order to predict the labels. If the accuracy of the classification is high, it means that our gene selection provides relevant information to separate the labels (phenotypic differences). For this purpose, we chose a Multi-Layer Perceptron Classifier, but it is possible to use other classification methods. First, the data set is split into training and test sets. The classifier is then trained and evaluated. Due to the stochastic training method, this process is repeated for a predefined number of sweeps. The output is the mean of the accuracy scores of all sweeps. If the classifier achieves a sufficiently high accuracy, and also compared to a model accuracy based on all available genes it does not have a significant drop, it means that the gene set captures enough information for a model to learn the relations between gene expressions and cell clusters. The reverse does not hold, as a poor model accuracy might be due to a suboptimal choice of model or hyperparameters for this purpose. In our case, we use the ML methods as an easy-to-use framework to generate a function connecting input with output and thus to prove the existence of such a function with sufficient accuracy based on our gene selection.

### Explainability

For further explainability of the resulting genes, we provide two ways of visualizing the impact of genes on the label decision. The first method utilizes the Shapley Additive Explanations (SHAP) [4] framework: https://github.com/slundberg/shap. SHAP is able to compute Shapley values from game theory based on a model and data. In our case, we train a Multi-Layer Perceptron Classifier using the genes detected by the pipeline as features. The SHAP KernelExplainer uses a special weighted linear regression in order to compute the Shapley values. The results are visualized for each possible label, please see the Results section for an illustration. The combination of the value on the x-axis and the color shows how gene expression influences the classification. The color implies if the corresponding gene is highly or lowly expressed. The values on the x-axis (SHAP values) show how much and in which direction the concrete value of the expression (encoded in the color) of the corresponding gene influences the classification for a certain single cell. A negative SHAP value implies that a gene’s expression level of the corresponding single cell supports the decision of the ML model against the respective label. A positive SHAP value implies the opposite, meaning that the corresponding expression value of the gene rather pushes the model to decide to classify the corresponding single cell into the corresponding class. The higher the absolute SHAP value, the higher the impact on the decision. If we next consider all single cells in the plot, we can derive some rules, like if gene X is highly expressed (many single cells/data points have a high value), then the model classifies a single cell to the corresponding phenotype (high SHAP value).

As an alternative second method, we train a Decision Tree. The tree structure, which represents the rules learned, is essentially based on two parameters: defining the maximum depth of the tree to be trained and the minimum number of samples required for the tree to form a new leaf. With these two parameters, we can prune the tree structure to the simplest one where classification still has sufficient accuracy. After the training, the tree structure is plotted. This allows for another approach to determine the relation between certain gene expressions and the classifier’s decision. The procedure works as follows: If the rule (expression of gene below a displayed threshold) is fulfilled, take the right branch, or else the left branch. By applying this instruction, we can construct relations about the expression level of genes and how these relations are connected to a label (phenotypic difference between two cell types). For an illustration, please see the Results section.

### Combinations of stages

**Fig 2:**
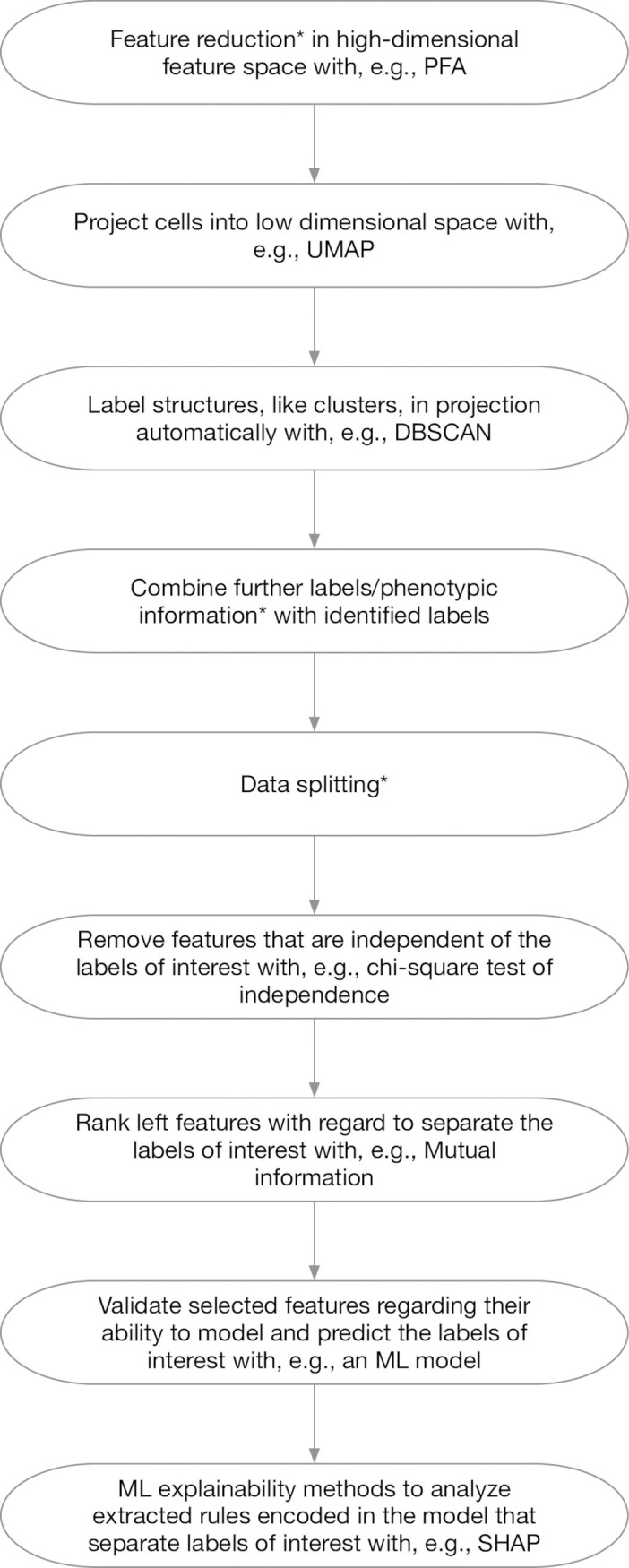
Schematic visualization of a possible sequence of steps performed during the analysis. Different combinations of the steps are possible depending on the data set and research question. The * indicates that stage is optionally and, depending on the data set and research question, might not be required to obtain a small and meaningful data set.

Of note, our pipeline (Fig 2) is designed to work even while skipping some steps, e.g., doing a UMAP plot without previous PFA dimension reduction. However, such a step can provide a different set of genes that are useful, e.g., in case no appropriate drug target is given in the provided set.

Furthermore, in our pipeline, the feature selection/reduction step before UMAP is not limited to PFA. Our modularized code can be easily extended with other methods. Even if the feature reduction generates a reduced space of transformed genes, like PCA or autoencoders do, we can switch back to the space of original genes after the labeling, apply PFA, chi-square test to identify label-related genes, and the validation and explainability, to get a small and meaningful (with high explanative power) set of genes from the original set of genes to have a well-interpretable result.

With this in mind, we describe the data preprocessing and the analysis of the biological data used to showcase the application of the characteristic gene extraction pipeline.

#### 1. Preparation

The preparation required for this analysis is a simplified and slightly modified version of the preparation steps required for the PFA described in Caliskan et al. (2023) [3]. In our showcase, we analyze the data set generated by Solé-Boldo et al. (2020) [6], which provides a Seurat object containing information on cell type and age group.

In the first preparation step (performed in R (version 4.2.0 [21]) using RStudio (“Prairie Trillium” Release (9f796939, 2022-02-16) [22])), we split the data set into subclusters according to condition (‘young’ or ‘old’).

The subsequent PFA requires only the gene names and the respective conditions, both of which are provided in the metadata of the Seurat object. The subsequent gene extraction steps are performed in Python; detailed information on the required packages for the preparation and version information can be found on GitHub (https://github.com/AC-PHD/NoLabelPFA, https://github.com/LauritzR/characteristic-feature-extraction). Our preprocessed file contained 21352 genes measured each in 15458 single cells.

As in our previously described simpler workflow requiring labeled groups and hence more information [3], the resulting selection is then transformed into an input table containing information on gene expression, the respective gene names, and labels according to the conditions.

For the subsequent analysis, the tool temporarily sets aside the gene names and labels and focuses solely on the counts data. Once the analysis is complete, the gene names and labels are reintegrated to determine the associations and characteristics of the different genes and single cells within the clusters.

The resulting output (preprocessed_data.csv, the gene expression data, and comparison_labels.csv, the information regarding labels to compare with results from clustering in stage 4) is required for the subsequent analysis.

#### 2. Analysis

**Fig 3:**
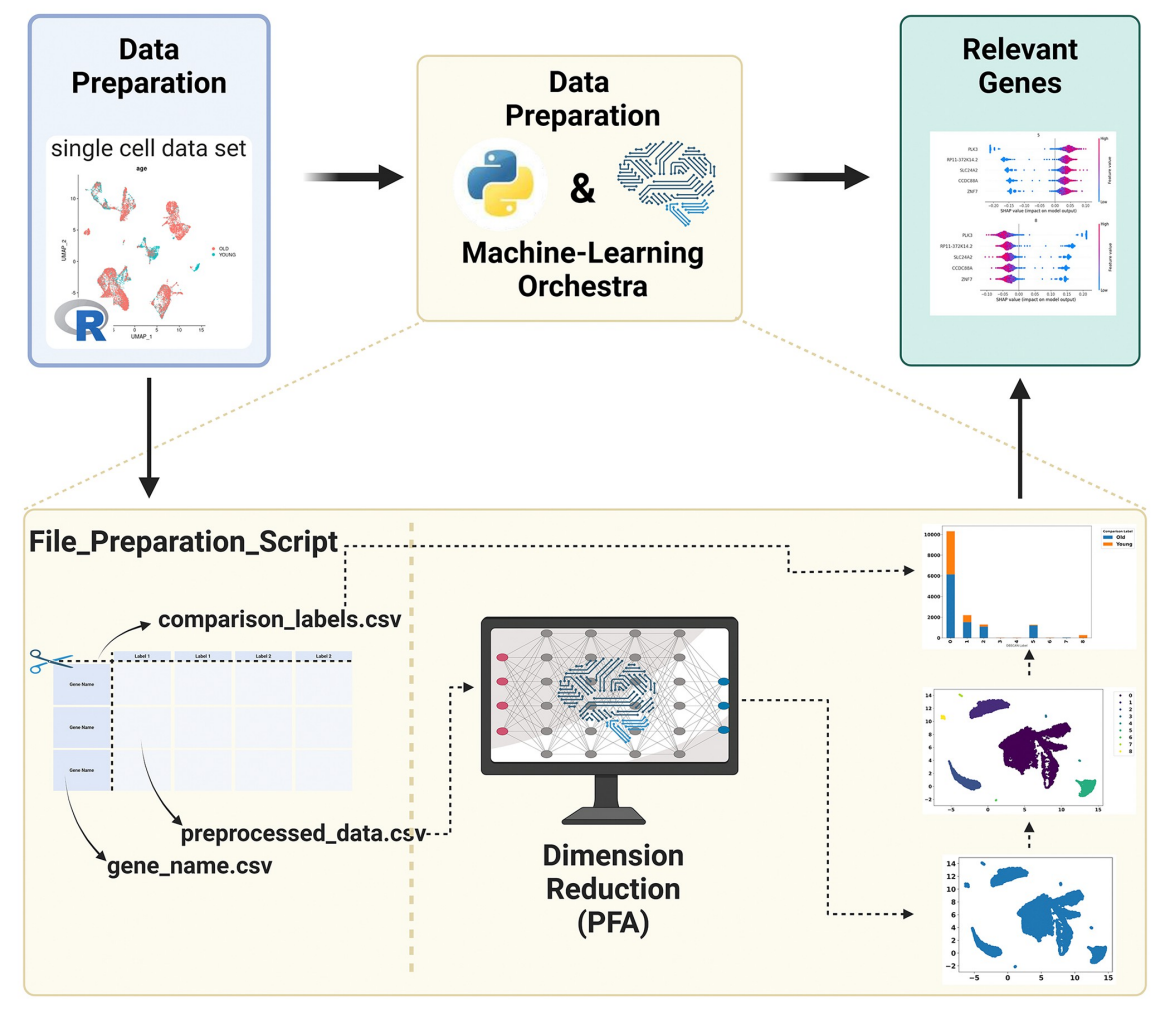
Visualization of the workflow extracting characteristic genes. Created with BioRender.com.

While in the workflow presented in Caliskan et al. (2023) [3] labels are required to find relevant genes characteristic for the differences, the new pipeline (Fig 3) is able to remove redundant genes without requiring the labels in advance. The reason is that the first part of the PFA does not require information like a label. For our example, we set the PFA parameters as cluster_size = 50 and min_n_datapoints_a_bin = 500.

The PFA results are used for a subsequent UMAP analysis, resulting in a UMAP embedding based on the reduced feature space.

Both DBSCAN and HDBSCAN can be used for the subsequent cluster analysis. For our analysis, we used the DBSCAN parameters eps = 1, min_samples = 15, and the HDBSCAN parameter min_cluster_size = 15. Ideally, these analyses result in several clusters containing only or mostly cells of one condition, even if the conditions of the original Seurat object overlapped since most conditions affect all cell types of a Seurat object. To select the clusters, the cell labels are reconnected with the respective cells and visualized as a bar plot containing a bar for each cell type, which is either split into two colors (for a “mixed group” of two conditions) or is (mostly) of a single color for “clean clusters” of a high purity.

By selecting “clean clusters” containing mostly cells of one condition (e.g., a cluster of mostly young cells and a cluster containing mostly old cells) and comparing these clusters directly with each other, the relevant genes for the different conditions are found. Therefore, the respective cluster numbers need to be selected for the “find_cluster_differences” steps, e.g., cluster 5, which contains mostly old cells, and cluster 8, which contains mostly young cells. In other words, we select genes by whose expression profile we can clearly assign the corresponding cell to a class. Since these classes coincide with the clear condition (here, a high purity of either young or old; see Fig 5 in Results), we can assume that the identified genes also encode important information for phenotypic differences.

Analogous to the previously described mutual information step [3], the get_mutual_information step selects and ranks the genes apparently responsible for the differences between the two clusters. We are aware that the pairwise mutual information between gene expression and label does not cover information that is captured in the combination of gene expression, e.g., two genes with each a low mutual information could have a big contribution to the label prediction. However, according to our experience, the pairwise mutual information is a purposeful approximation that provides working examples in a short computation time. An alternative is to perform the validation step on each combination of genes, e.g., after or even before the find_cluster_differences, to find a combination of genes based on whose expression values a classification with a sufficient accuracy can be made. However, such an approach might be hindered by the combinatorial effort. While only the top-ranked gene would have been sufficient for separating the clusters correctly, we decided to consider the five top-ranked genes for the next analysis steps, which gives more context and resulted in an accuracy of 100%. Therefore, the parameters were set to select five genes (n_highest_mutual_information = 5) and analyze the difference between clusters 5 and 8 (clusters = [8,5]). For the tree explainer, we have chosen min_samples_leaf = 10. To evaluate the impact of the respective genes on the decision using SHAP, we applied the same parameter settings (n_highest_mutual_information = 5, clusters = [8,5]).

The validation step gives information on the correlation between the number of selected genes and the resulting prediction accuracy (similar to the validation in Caliskan et al. (2023) [3]).

#### 3. Enrichment analysis

To showcase a possible downstream analysis method, we performed an enrichment analysis in R by analyzing the five top-ranked results using the clusterProfiler Package [23, 24] (version 4.4.2) and the C5 ontology gene set of the Molecular Signatures Database (MSigDB) [25, 26]. The C5 collection contains various ontology gene sets, which can be divided into Gene Ontology Biological Processes (GOBPs), Gene Ontology Cellular Components (GOCC), and Gene Ontology Molecular Functions (GOMF).

## Results

**Fig 4:**
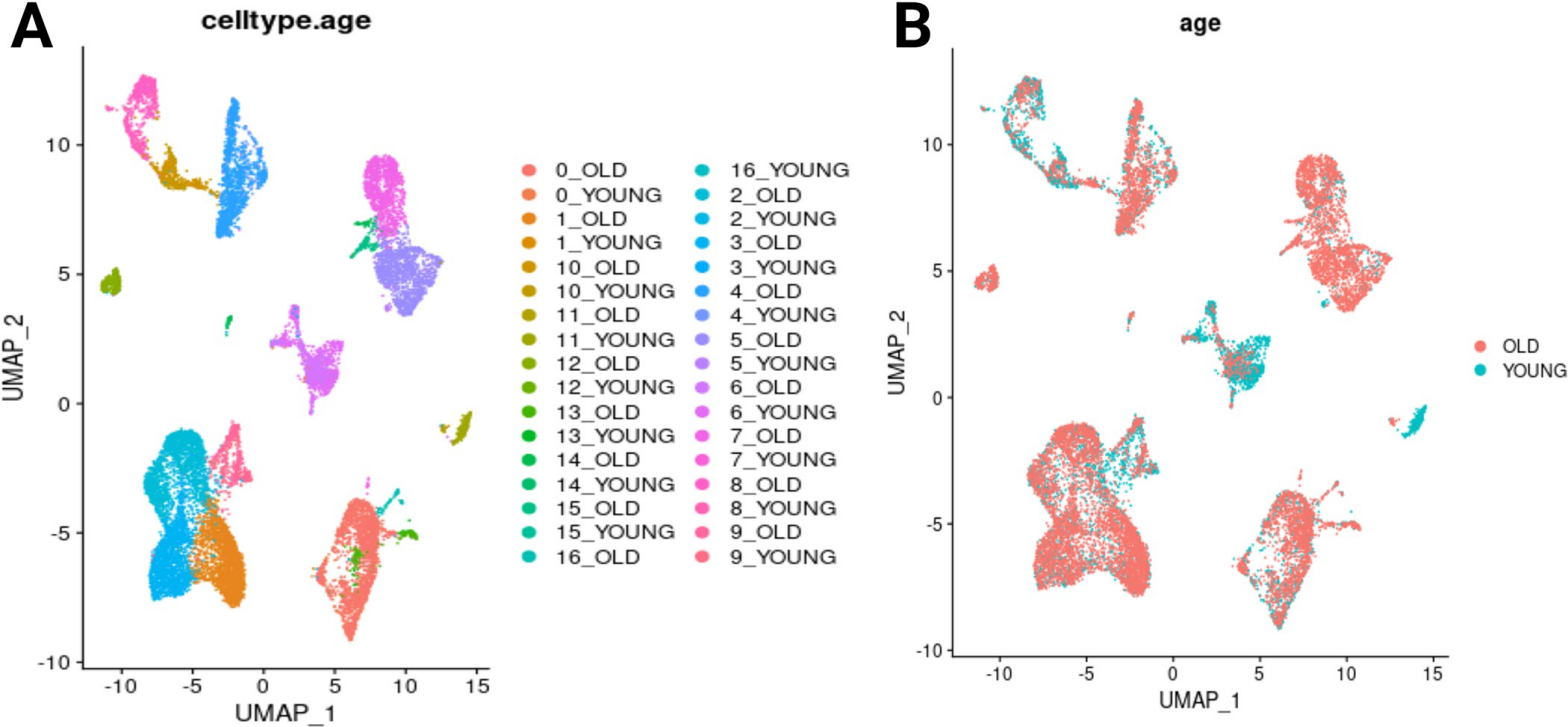
Clustering of the Seurat object. (A) Clustering by cell types and ages. (B) Clustering by age.

**Fig 5:**
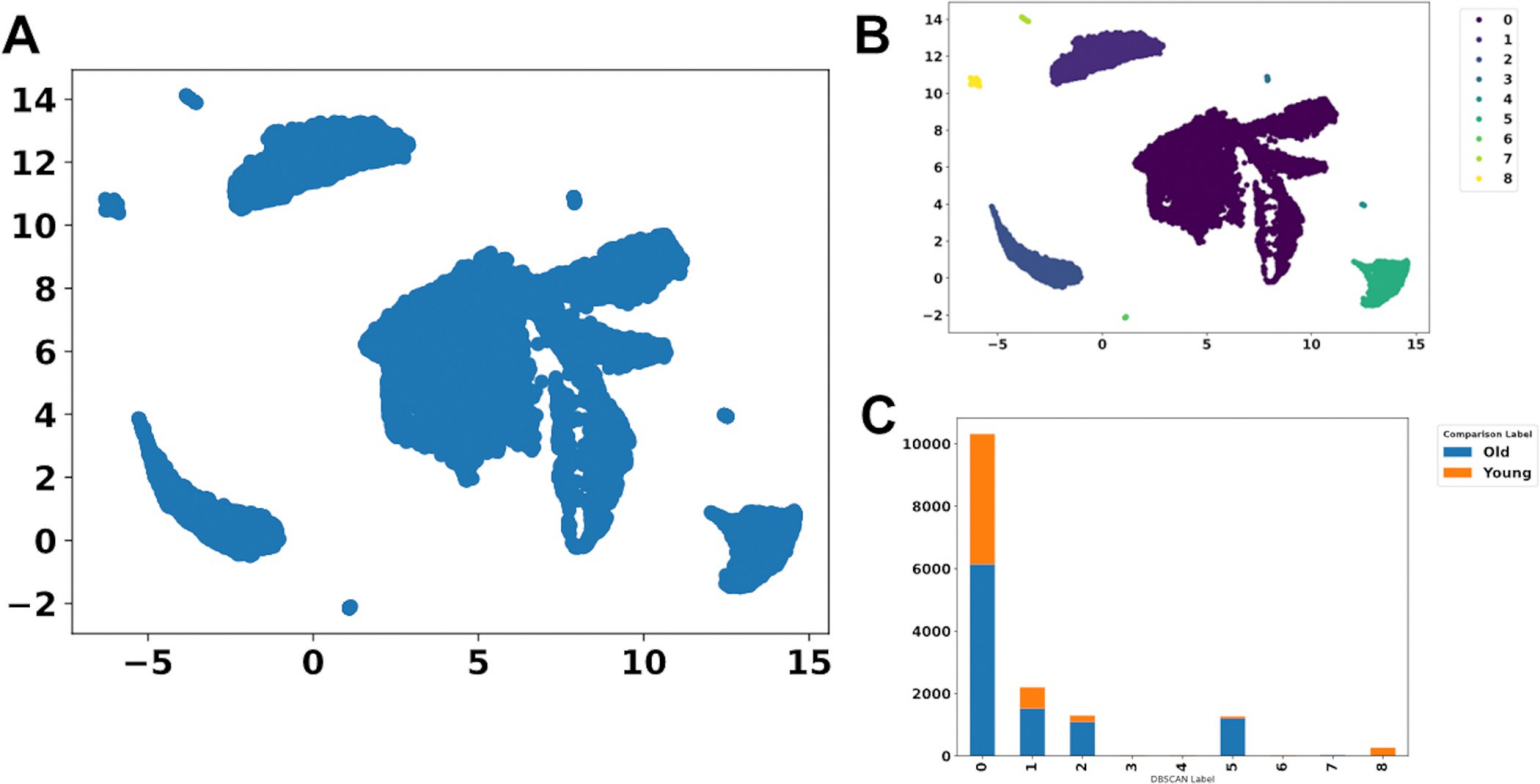
Visualization of the selected PFA genes. (A) UMAP plot of the relevant PFA genes. (B) Clustering according to DBSCAN. (C) Composition of the different clusters according to condition (OLD or YOUNG).

Our pipeline delivers its results in the following order:

- unlabeled feature selection determines objectively groups, including minimal gene sets separating different groups (clustering of Seurat objects; Fig 4)
- Next, ML explainability methods transform the features correlating with phenotypic differences into causal reasoning.
- This is further supported by additional pipeline and visualization tools (Fig 5), allowing user knowledge to be integrated, which further boosts causal reasoning and explainability (Figs 7 and 8).

The following section examines in detail the results achieved by the pipeline, integrating all three steps. The potentially relevant genes resulting from the analyses of this pipeline are visualized in Fig 7 and available in the S1 Data. Additionally, the complete results of this analysis are available in the S1 Data. Users interested in more than the five top-ranked genes need to adjust the value of “n_highest_mutual_information” accordingly.

The clustering of the Seurat object of the data set by Solé-Boldo et al. (2020) [6] (source of the data: https://www.ncbi.nlm.nih.gov/geo/query/acc.cgi?acc=GSE130973) in R shows that Seurat objects of single-cell sequencing data usually cluster by cell type (Fig 4). While the cell types are clearly separated, almost all cell types appear to contain cells of both conditions (OLD and YOUNG).

For clusters that show no clear differences between two conditions (e.g., treated vs. untreated, different tumor types, or, as in our workflow for demonstration purposes, ‘old’ and ‘young’ cells), our showcased method follows a different approach. The first step of the workflow is the previously described PFA [3], which removes redundant genes, resulting in a smaller set of genes (keeping only 5413 genes of the 21351 genes), which are subsequently visualized as a UMAP plot (Fig 4A).

The UMAP plot of the relevant PFA genes shows clear clustering (Fig 5B, visualized with DBSCAN). While some of these clusters contain cells of both conditions (e.g., cluster 1 in Fig 5C), two clusters contain mostly cells of only one condition (e.g., cluster 5 and 8 in Fig 5C). If the bars indicate the presence of more than one cell type, this might be due to the cell types not being clearly separated by gene expression, and the differences in phenotype might be due to other differences between the conditions, such as epigenetic effects like methylation or other information that is not encoded in the current gene expression data set. Furthermore, a different hyperparameter setting of the used embedding method, the feature selection method, or if one is used at all, could also influence how single cells are clustered such that clusters coincide with certain conditions. In the present case, clusters 5 and 8 contain a population of high purity from each condition based on the methods and hyperparameters used.

**Fig 6:**
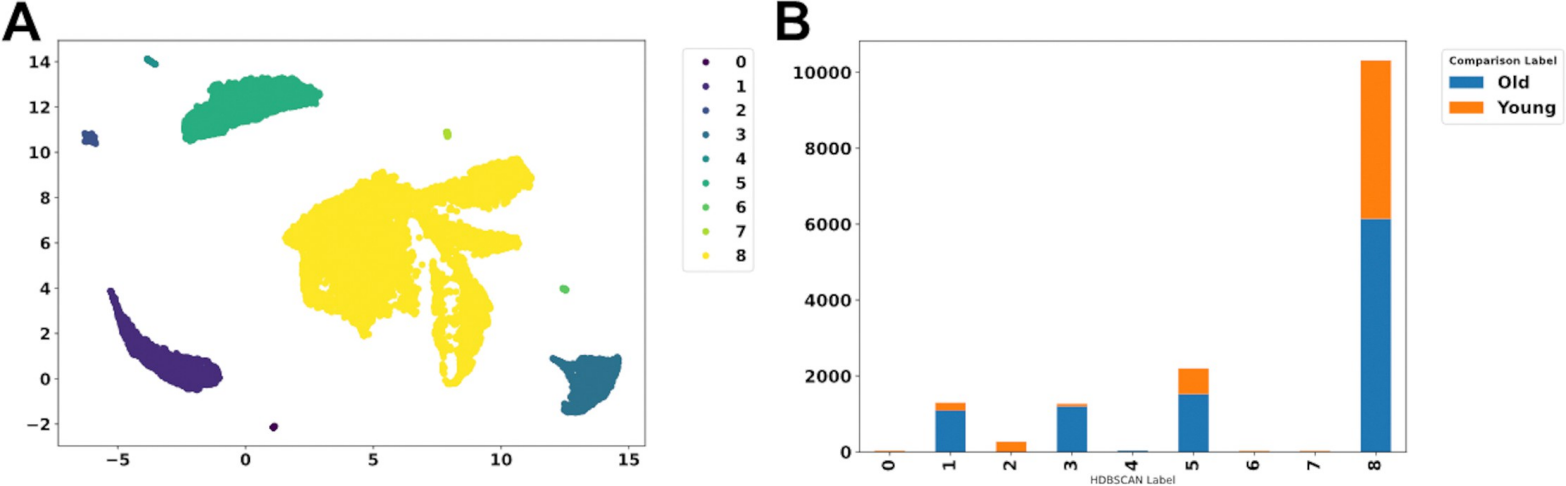
HDBSCAN can be used as an alternative to DBSCAN. (A) Clustering according to HDBSCAN. (B) Composition of the different clusters according to condition (OLD or YOUNG).

As an alternative to DBSCAN, it is also possible to use HDBSCAN, which also results in clearly separated clusters (Fig 6A), which contain cells of both conditions (cluster 8 in Fig 6B) or mostly cells of one of the conditions (e.g., cluster 2 and 3 in Fig 6B).

Visualization of clusters that are separated by the condition is an advantage of the compared pipeline compared to the overlapping visualization of the conditions of the original Seurat object (Fig 4B), which was specifically chosen for this showcase. For the validation step, we have set the number_sweeps = 20. Choosing five genes resulted in a balanced accuracy of 100% for the training data and a balanced accuracy of 100% for the test data. If we choose five random genes in each sweep, we obtain a balanced accuracy score of about 52% on the train and test set. This clearly shows that a selected small number of genes carries precise information to separate the classes that deviate from randomness. As a control, we validate on all available genes that have a non-constant expression level, providing also a balanced accuracy of 100% on the train and test set. The high accuracy both on all genes and our selected ones shows that the five genes selected by our pipeline contain all the relevant information from the total data set with regard to separating the corresponding clusters.

**Fig 7:**
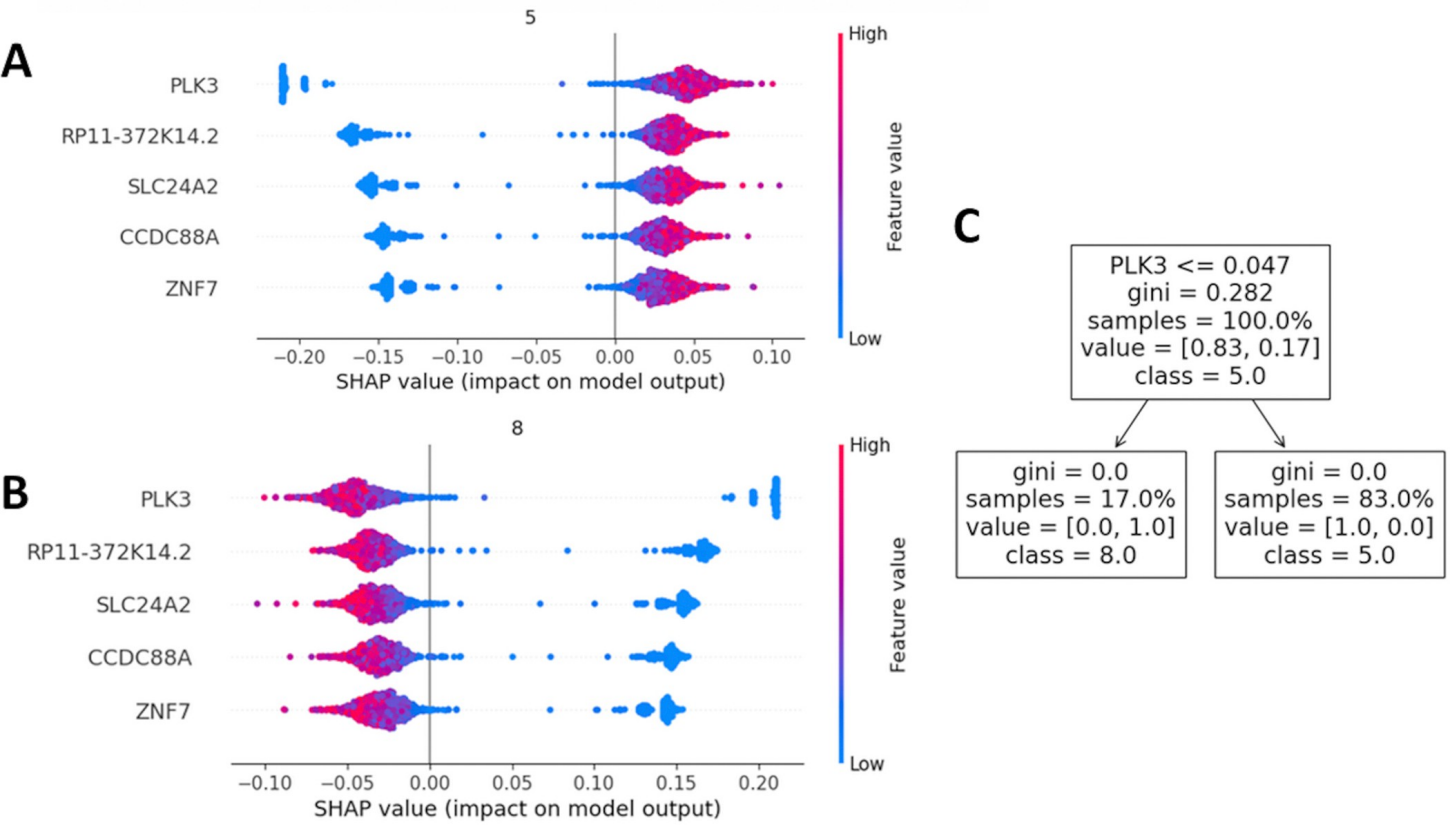
Gene expression and condition of the five top-ranked results. (A) SHAP values of cluster 5 (mostly old cells; compared to the young cells, the gene expression is upregulated in the old cells). (B) SHAP values of cluster 8 (mostly young cells; compared to the old cells, the gene expression is downregulated in the young cells). (C) The tree explanation visualizes the top-ranked result (in this showcase PLK3) and the clustering for PLK3 expressing samples. 17.0% of the PLK3 expressing cells are among the cells of cluster 8 (mostly young cells), and 83.0% of the PLK3 expressing cells are among the cells of cluster 5 (mostly old cells). Thus, PLK3 is upregulated in old cells.

For the subsequent analyses, the results of the DBSCAN step (Fig 5B) were used, specifically, cluster 5 (mostly old cells, see Fig 5C) and cluster 8 (mostly young cells, see Fig 5C). In the explainability analysis (Fig 7), we see that high expression of the characteristic genes is a clear separator between fibroblasts from young and old humans since, in Fig 7A, high expression values make the model decide to classify a single cell into class 5 (condition “old”), which is what the positive SHAP value means (x-axis). In addition, in Fig 7C (tree explainer), we see that a decision for class 8 is based on the rule if the expression value of PLK3 is below 0.047. If this is the case, a cell is classified with label 8 (condition “young”). If the expression value is above 0.047, the decision tree decides for class 5 (condition “old”).

**Fig 8:**
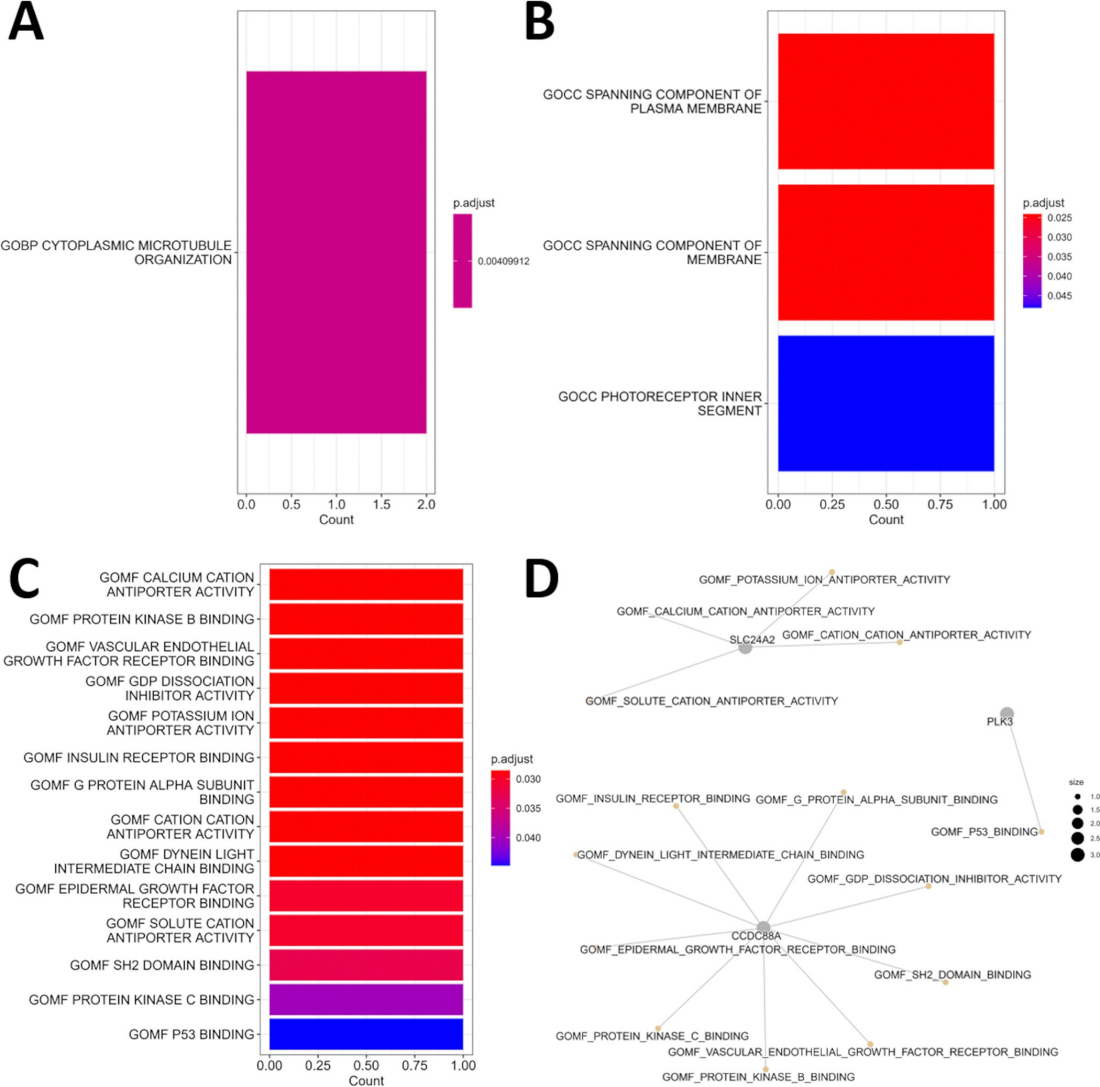
Gene ontology enrichment analysis of the five top-ranked genes. The color gradients in A to C indicate the adjusted p-values. (A) The resulting Gene Ontology Biological Processes (GOBP). (B) The resulting GO Cellular Components (GOCC). (C) The resulting GO Molecular Functions (GOMF). (D) CNET plot visualizing the GOMFs that were associated with the five top-ranked genes.

Additionally, we performed an enrichment analysis using the five top-ranked genes. This step only requires a list of the respective gene names as input, and the enrichment results can help estimate the top-ranked genes. Since Solé-Boldo et al. (2020) analyzed single-cell data derived from healthy donors of two age groups (“young” and “old”) [6], the results of the enrichment analysis (Fig 8) ought to be associated with age-related changes. The most relevant genes are associated with a biological process (“CYTOPLASMIC MICROTUBULE ORGANIZATION”), three cellular components (“SPANNING COMPONENT OF PLASMA MEMBRANE”, “SPANNING COMPONENT OF MEMBRANE”, and “PHOTORECEPTOR INNER SEGMENT”) and 14 molecular functions, including “VASCULAR ENDOTHELIAL GROWTH FACTOR RECEPTOR BINDING”, “EPIDERMAL GROWTH FACTOR RECEPTOR BINDING”, “P53 BINDING”, and “INSULIN RECEPTOR BINDING”. Some of the results are associated with the cell cycle, or cellular senescence, which is among the Hallmarks of Aging [27, 28]. Additionally, some of the results can be associated with other Hallmarks of Aging [27, 28] or the Hallmarks of Fibroblast Aging [12].

## Discussion

### The novelty of the approach and comparison to alternatives

Our analysis resulted in a ranked list of several genes that appear to be essential for the correct prediction of the condition, meaning that the separation of expression patterns indicates a clear characteristic difference between young and old cells. Despite the original data showing no clear clustering regarding the condition (young/old) due to the experimental setup for our use case, the analysis steps resulted in several clearly separable clusters, containing mostly young or mostly old cells, as well as “mixed clusters” containing cells of both conditions.

Since we compared ‘young’ and ‘old’ using all genes of the data set, the expression of the single-cell genes of the clearly separated clusters of high purity of either young or old should be involved in the age-related differences between all of the cell types. As demonstrated above, the PFA approach in combination with DBSCAN (and alternatively HDBSCAN) was able to obtain genes relevant for clustering according to condition and resulted in clusters containing mostly cells of the same condition.

This showcases that our presented pipeline can separate cells of different conditions into separate clusters for each condition and identify characteristic genes for the differences. As a new supplementary tool, this approach allows the researcher to focus on the general differences between the single-cell data of different conditions, in particular where conditions such as a phenotypic property are given via additional labels, which can be infused into the label comparison step.

We remark that a comparison of methods in the area of causal reasoning, both quantitatively and qualitatively, is challenging: First, in nature, there are several explanations possible for the same observation, e.g., depending on where one starts explaining. Second, if a phenotype of a cell depends on the activation of two pathways with each several genes, one by one organized in each pathway, then the expression profile of both pathways can be described by a pair of genes, one gene from each pathway. This example demonstrates that any pipeline providing a small and minimal gene expression set cannot provide a unique solution in such a case, while all results preserve all necessary information to explain the total gene expression of both pathways. Third, since the former example demonstrates the issue of identifying a ground truth for a difference and thus several equal solutions can co-exist, a quantification of the accuracy of different pipelines is also challenging.

Hence, we make no direct comparison of different tools compared to our pipeline but rather aim at providing an additional view of the data with our tool, getting another set of genes from which an explanation might come more easily. The presented pipeline identifies a mathematical ranking of genes in the separating gene set.

Similarly, we do not favor any particular method for causal reasoning but rather believe that our pipeline allows using an array of methods to give a strong basis to determine which causal relations are there. Besides ML methods, we also actively incorporate user-specific domain knowledge via our visualization tools.

In the following, we would like to show how our method differs from existing methods in terms of mathematical methods since different methods rely on different assumptions that might be more fulfilled on some data sets and on others less such that we get different results with each method. Even if each result captures all necessary information for describing an observation (assuming corresponding assumptions are sufficiently fulfilled), depending on the experience of the users, some might find an explanation more easily from one result and some from the other.

### Related methods

Hancer et al. (2020) have reviewed various methods employing unsupervised learning and unsupervised feature selection [29]. They highlight the importance of clustering as a typical part of unsupervised learning, focusing on feature selection and describing and evaluating a variety of different feature selection approaches [29]. Several of these approaches combine the selection of features and clustering [29]. Nevertheless, all of these approaches have some drawbacks. For instance, some require all features during the validation of the clusters despite having selected a number of the most relevant features, while other promising approaches combining clustering and eliminating redundant features have not been thoroughly experimentally assessed [29]. Additionally, some approaches require that the number of clusters or features is predefined; others do not perform proper clustering for data with unshaped (non-gaussian) distribution, which is common in real-world data [29]. While approaches employing sparse learning give promising results, they also require computational power and complex matrix operations, and Hancer et al. (2020) emphasize the need for hybrid approaches integrating filters to reduce the complexity of the data and wrappers for finding feature subsets [29]. Our work aims to provide a framework combining different techniques with the focus of finding explanations for phenotypic differences originating in the gene expression or in the input feature set, respectively. Due to our modularized code architecture, provided via a GitHub repository, our pipeline can be easily extended.

Shi et al. (2023) propose a novel approach to mitigate the challenges of unsupervised feature selection, which often leads to information loss when relying solely on pseudo labels or no labels at all. Their method leverages binary hash codes as weakly-supervised multi-labels, which are autonomously learned to enhance the accuracy of feature selection [30].

While introducing labels during feature selection can improve the accuracy, our approach differs in that it facilitates user interaction with the data and helps infuse their domain knowledge into the labeling process, which marks interesting information for causal relations.

Other approaches, such as the unsupervised feature selection using flexible optimal graphs (FOG-R and FOG-C), recently introduced by Chen et al. (2022), aim to improve and optimize the similarity matrix used for feature selection, resulting in a sparser projection matrix and a better performance than other current unsupervised feature selection algorithms [31].

The process of feature selection is also being optimized, for instance, in the approach by Li et al. (2022): multiple feature filters and single common feature filter [32]. Their unsupervised 2-D weight-based approach eliminates the need for additional hyperparameters and surpassed contemporary methods [32].

Another optimization approach is to minimize redundancy, for example, by using Gong et al.’s autoencoder with redundancy control (AARC) [33] or the principal feature analysis (PFA) [3]. These approaches assume that features can correlate or be dependent and thus can be regarded as redundant. Reducing this redundancy can improve the network structure and increase efficiency [3, 33]. Since all of these methods reduce the feature space according to different mathematical rules based on different assumptions to work properly, the selection of a feature selection method might depend on the concrete data set to provide remaining features that allow a powerful causal explanation. In order to lower the effort to include further methods, our GitHub repository is structured in a modularized way, allowing an easy exchange of the feature selection method that seems most reasonable to the user while nothing has to be changed in the subsequent pipeline.

The variety of unlabeled clustering approaches has been discussed in detail by Hancer et al. (2020) [29], and more recent approaches, such as those introduced by others, including Shi et al. (2023) [30], Gong et al. (2022) [33], and Chen et al. (2022) [31]. Our method is unique so far in terms of the combination of methods that are intended to provide detailed checkpoints by visualization in the data processing from raw single-cell data to an explanation of phenotypic behavior. At each step and checkpoint, the user can infuse their domain knowledge about biology and can actively control the intermediate results to optimize causal explanations.

We highly appreciate the many approaches that have been developed and suggest combining different approaches in a similar manner as different data sets require different methods that fit better to the given data structure. In our example, selecting only one gene would have been sufficient for discerning between both clusters and using five genes resulted in a prediction accuracy of 100%. Since these genes appear to be essential for the correct clustering, they might be relevant for the differences between the two conditions–in our example, the differences in skin fibroblasts of a typically sun-protected area between younger and older men whose separation of cells highly correlate with the two clusters investigated. As discussed below, all of these five genes can be linked to aging, although they are not the first genes that come to mind when thinking about aging. This demonstrates that our approach is a valuable additional method for identifying potentially relevant genes, even with a ranking, possibly resulting in genes that might be of greater relevance than currently assumed and thus resulting in a better allocation of resources.

### Critical evaluation of the achieved biological results

By comparing a cluster containing mostly young cells with a cluster containing mostly old cells, the results of our showcase analysis should reveal age-related changes. However, the method is also suitable for other analyses, e.g., for comparing treated and untreated cells or resistant vs. responding tumor cells.

The resulting ranked list of relevant genes is based on the mutual information in the analysis [3]. However, we combine here in our new powerful pipeline: (i) unlabeled feature selection, (ii) ML explainability methods transforming features correlating with phenotypic differences into causal reasoning, with (iii) further pipeline and visualization tools.

With this optimized pipeline, several biological implications can be derived by using our pipeline directly on the single-cell sequencing data without further analysis and with a focus on assessing individual high-ranked genes with the best information:

Since this approach pooled all single cells of the younger donors in one group and those of the older donors in a second group, both groups contain several cell types. The changes in gene expression will therefore reflect the changes that were deemed most relevant for the differences between the two age groups according to the ML approach.

While it is possible that not all cell types within the two groups express all these genes, the most relevant genes, ranked by mutual information, enable the algorithm to separate the cells according to their cluster-belonging. However, since the clusters also have a high purity of properties, young and old, the difference between these clusters should encode for general expressional differences of aged cells.

According to the SHAP results (the ML explainability algorithm we used [4]), all of the five top-ranked results (PLK3, CCDC88A, ZNF7, SLC24A2, and RP11-372K14) in the mutual information were upregulated in the old cells, indicating that these genes might be relevant for age-related changes. When analyzing new data or in a study examining age-related changes in gene expression, these genes would now become the focus of further attention and subsequent analyses. To demonstrate the relevance and significance of our methodology, we will now assess these five highest-ranked potential genes of interest and their relation to aging.

The top-ranked Polo-like protein kinase 3 (PLK3) was deemed as the most relevant gene by our ML method and appears to be upregulated in old age.

Although PLK3 is the least explored polo-like kinase [34], it might be relevant for aging, as it is one of the 55 core senescence genes reported by Hernandez-Segura et al. (2017) [35]. In their analyses, PLK3 was among the upregulated genes associated with G1 DNA damage checkpoint and regulation of the mitotic cell cycle [35]. Therefore, upregulation of PLK3 in the old single cells might indicate a higher number of senescent cells in the old samples, which corresponds with the well-known age-related increase of senescent cells and senescent fibroblasts in aging skin, which further contribute to aging and inflammation via the senescence-associated secretory phenotype (SASP) [36]. Since upregulation of PLK3 is associated with DNA damage, DNA damage could be a causal relation that drives the aging process. If DNA damage is a cause for aging in fibroblasts, it is necessary that a measure of DNA damage in the cells of the cluster with cells from the young donors is smaller than in the cluster with the cells obtained from the older donors. A measure for DNA damage could be, e.g., the number of sites where the DNA has mutations. This example demonstrates how such an analysis can guide useful experiments data-driven from the single-cell data to validate the hypothesized relations targeted. Furthermore, it can be seen as a guided data collection as DNA damage can be seen as a feature additional to the gene expression profile.

Due to the link between aging and inflammation, often referred to as “inflammaging” [37], inflammation is among the critical areas of aging research defined by Kennedy et al. (2017), which are also referred to as the seven “pillars of aging” [38] and are part of the expanded “Hallmarks of Aging” [28]. Other areas of interest in aging research include but are not limited to cellular senescence [27, 28], genomic instability [27, 28], the accumulation of DNA-mutations [27], and adaption to stress [38].

PLK3 has also been associated with DNA repair [34] and has been examined regarding its function in the response to different types of cellular stress and in oncogenesis [34, 39, 40]. In aging mice, for example, Plk3 deficiency (in Plk3-KO mice) appears to accelerate tumor development and results in more pronounced angiogenesis and larger tumor size, indicating a tumor-suppressing function [41]. In human cells, PLK3 has garnered interest for its possible effect on the treatment response in colon carcinoma, prostate cancer, and melanoma [34].

Due to its participation in the stress response upon environmental stresses and oxidative stress, it is assumed that PLK3 might be mainly involved in regulating the stress response, and the inhibition of PLK3 has been shown to attenuate injury-induced apoptosis in a mouse model of renal ischemia-reperfusion (I/R) injury [42]. However, besides regulating DNA damage response and apoptosis, PLK3 has also been linked with cell cycle progression by regulating S phase entry [39, 41] as well as centrosomal function and the regulation of microtubule dynamics, with its dysregulation resulting in cell cycle arrest and apoptosis [43].

Consequently, an in-depth investigation of PLK3 and its regulation during the aging process might lead to a better understanding of the aging process and potential anti-aging interventions, making PLK3 an interesting target for subsequent laboratory research.

The second-ranked gene, Coiled-Coil Domain Containing 88A (CCDC88A), has also been associated with cancer and aging [44, 45] and is involved in various biological processes, including tumor angiogenesis, cancer migration and invasion, tumor metastasis, and epithelial wound healing [44].

It is also known as Girdin (G alpha-interacting, vesicle-associated protein) and GIV and interacts with STAT3 [46] and AKT [44].

The transcription factor signal transducer and activator of transcription-3 (STAT3) is a central regulator of metastasis and is known to regulate genes that are involved in cancer and wound healing [46]. Additionally, STAT3 has been reported as promoting a youthful epigenetic state in articular chondrocytes [47], and interacting with STAT1 and thereby regulating senescence and inflammation, which might indicate its potential as a therapeutic target for treating the senescence-associated inflammatory phenotype in obesity-related type 2 diabetes [48].

In cancer and wound healing, STAT3 directly targets and upregulates CCDC88A, which also enhances the activation of STAT3 via a positive feedback loop [46]. The upregulation of CCDC88A during cancer progression and wound healing has been reported as being essential for cell migration during both processes [46].

Since wound healing is impaired and delayed in elderly patients, understanding the age-associated changes has been of great interest [49]. Due to CCDC88A being involved in wound healing and interacting with STAT3, which appears to affect inflammation, examining the role of CCDC88A in aged skin and during wound healing in aged skin might allow further insights into age-related changes and impairments.

Vu et al. (2022), who analyzed single-cell sequencing data of young and aged mice and compared wounded and unwounded skin, report that fibroblasts in unwounded skin only showed minor age-related changes [49]. Therefore, observing the effects of different CCDC88A expression levels in wound healing assays in skin samples of donors of different ages might be of interest in subsequent analyses.

Besides being of interest due to its involvement in cancer and epithelial regeneration/repair [46], the role of CCDC88A in cell aging has also garnered interest due to its function as a direct downstream mediator of Akt signaling [45]. Lan et al. (2021) observed in human endometrial microvascular endothelial cells (HEMECs) that the activation of CCDC88A, which is mediated by Sirtuin 1 (SIRT1) and its deacetylation of Akt and PDK1, which subsequently activate CCDC88A, results in delayed aging [45]. Both SIRT1 and Akt are involved in aging and have already been shown to play essential roles in HEMEC aging [45]. SIRT1, along with all Sirtuin isoforms, for example, is well-known as an attractive therapeutic target for aging-related diseases and for its activation by the Sirtuin activating compound resveratrol, which can be found in grapes and red wine [50]. Lan et al. (2021) suggest that SIRT1, Akt, and CCDC88A might be potential therapeutic targets to treat HEMEC aging [45].

The ranking of CCDC88A as the second-most relevant gene in our showcase-aging-analysis indicates that the ML method can contribute to existing analysis methods while also highlighting the potential importance of CCDC88A in aging.

Additionally, CCDC88A has been linked to brain tumor stem cell stemness, mTORC1 signaling, DNA damage-induced cancer cell apoptosis, and cell cycle progression and appears to affect the sensitivity of cancer cells to therapeutics [51]. Since CCDC88A appears to be essential for glioblastoma migration and invasion, and is associated with glioma malignancy, it has also been suggested as a novel therapeutic target in malignant glioma [52]. Therefore, further laboratory analyses regarding its expression and regulation upon aging are of great interest.

Like CCDC88A, the third-ranked result, Zinc Finger Protein 7 (ZNF7), has been associated with glioblastoma [53]. The C2H2 ZNF protein ZNF7 is a member of the transcription factor subfamily Krüppel-associated box (KRAB) ZNF subfamily of ZNF transcription factors, which are characterized by having several C2H2 ZNF motifs and a KRAB repressor domain [53]. Despite being the largest family of transcription factors in higher eukaryotes, there is still little known about the C2H2 zinc finger proteins [53, 54]. However, some of them have been associated with important functions during embryonic development as well as in cell cycle regulation, cell proliferation, cell differentiation, and apoptosis [53]. In glioblastoma, ZNF7 has been reported as a survival marker [53]. While Esteve-Codina et al. (2021) report that longer survival was associated with a high *ZNF7* RNA expression, they also remark that further analyses need to be performed, as a ZNF7 variant could not be detected with the antibodies used in their analyses [53]. Additionally, *ZNF7* has previously been reported as upregulated after the induction of apoptosis [55] and has been associated with translational regulation and a potential role in systemic autoimmune arthritis [56]. Although ZNF7 has not explicitly been associated with aging yet, our results and its known associations indicate that further research regarding its role and its potential functions in aging is warranted.

The fourth-ranked result, the upregulated solute carrier family 24 member 2 (*SLC24A2*), encodes the K^+^-dependent Na^+^/Ca2^+^ exchanger NCKX2, which is a member of the NCKX family and appears to be vital for motor learning and spatial working memory [57]. In a rat model of chronic constriction injury (CCI), overexpression of *SLC24A2* reduced the expression of inflammatory cytokines (tumor necrosis factor‑α and interleukin (IL)‑1β, and IL‑6) and appeared to reduce pain [57]. Zhou et al. (2020) report that an increased expression of microRNA (miR)‑135a‑5p upon CCI downregulated *SLC24A2*. This results in a decrease of *SLC24A2* and NCKX2, which appears to be involved in the progression of neuropathic pain (NP), a long-lasting refractory disease that often results from peripheral nerve injury and occurs in up to 10% of the general population [57]. NP is frequently observed in patients with AIDS, cancer, lumbar disc syndrome, or trauma [57]. Since selective regulation of NCKX2 expression or miR-135a-5p could relieve neuralgia, miR-135a-5p and its target, *SLC24A2*, might be therapeutic targets for treating NP [57]. Additionally, miR-135a has been associated with nervous system diseases, including malignant glioma, Alzheimer’s disease [57], which was placed sixth among the leading causes of death for 2019 [58], and Parkinson’s disease [57], which is the second most common neurodegenerative disease after Alzheimer’s disease [59]. In skin, *SLC24A2* has been reported to be associated with skin color variation and it has been hypothesized that it affects melanocyte stem cells, which might cause changes in skin color, and might be involved in stress-related hair graying [60]. That it is among the top results of our showcase analysis comparing ‘young’ and ‘old’ single cells also suggests the possibility of *SLC24A2* being involved in age-related skin changes and warrants further laboratory research.

Our mathematical approach allows us to identify differences between two conditions, such as age-related changes in this showcase. This approach is not limited to genes but can also detect changes in long non-coding RNAs (lncRNAs).

The fifth-ranked result, the upregulated RP11-372K14.2 is among the top five results of the ML-analysis. First of all, this demonstrates that, of course, we can compare here, besides protein expression via mRNA, any separating feature such as lncRNAs (or, for instance, metabolite data or other features present).

Regarding the specific lncRNA, it has also been reported as one of the top five upregulated lncRNAs in coronary artery disease (CAD) compared to healthy samples by Zhang et al. (2021) [61] and might also be involved in chronic obstructive pulmonary disease (COPD) [62]. It appears to be regulated by Forkhead box A1 protein (FOXA1), which has been associated with the activation of p16^INK4a^ during cellular senescence [63]. Thus, it might also be involved in aging or age-related changes and pathologies.

All of the five top-ranked results of our ML analysis can be associated with aging by comparing ‘young’ and ‘old’ cells, which indicates that the ML method applied in this analysis can successfully indicate potential targets for aging research. Thus, this “first glance at potential research targets” offers a convenient and efficient additional analysis approach for researchers. This shows the potential use of our ML method as an additional approach for indicating potentially relevant target genes starting from less distinctly separated clusters.

### Embedding for higher dimensions than two

Besides the fact that not all information responsible for phenotypic differences might be encoded in the gene expression data (e.g., methylation), some clusters might only be separable in higher dimensions than two. For this purpose, the UMAP provides the option to also embed in three or higher dimensions (but low dimension compared to the dimension of the gene expression vector space) by the parameter n_components. Visualization might be challenging in this case of more than three dimensions; however, the DBSCAN or HDBSCAN can still be applied. Consequently, one can always check in the “compare labels” step (stage 4) and see if, e.g., clusters have been identified with a high purity of a phenotypic marker/property that is worth analysis of the characteristic differences.

### GO enrichment analysis

The result of the GOBP enrichment analysis, the GOBP Cytoplasmic Microtubule Organization, based on our highest mutual information-ranked genes, indicates that the microtubule might be affected by age-related changes. Microtubules play an important role in the formation of the spindle apparatus, also known as mitotic spindle, which is vital for cell division [64] and thus the cell cycle. Besides being involved in cell migration, proliferation and differentiation, microtubules also play a part in mitochondria transfer, during which they are cross-linked with mitochondria via dynactin/dynein and kinectin/kinesin [64]. Additionally, the GOMF Dynein Light Intermediate Chain Binding is also among the enrichment results and has been associated with CCDC88A, which is also associated with the GOMF Insulin Receptor Binding.

The impairment of the insulin receptor’s insulin binding ability, which is associated with disruption of the brain glucose homeostasis and brain aging, as well as peripheral insulin resistance, which is known as a typical feature of older age, highlight the importance of proper insulin signaling and insulin sensitivity [65]. As a maintained insulin sensitivity has been reported in the population of the oldest-old, while insulin resistance has been linked with an increased risk for cognitive decline and dementia, including Alzheimer’s disease [65], Insulin Receptor Binding could be of great interest in aging research. This is also confirmed by the association of insulin sensitivity/reduction of insulin resistance as a favorable outcome for interventions targeting the Hallmarks of Aging “Disabled macroautophagy”, “Deregulated nutrient-sensing”, and “Mitochondrial dysfunction” [28].

The vascular endothelial growth factor (VEGF) signaling family has also been associated with Alzheimer’s disease [66], indicating a possible connection between the GOMF Vascular Endothelial Growth Factor Receptor Binding and age-related disease, especially since the vascular endothelial growth factor has already garnered interest as a potential therapeutic opportunity for the treatment of Alzheimer’s disease [67]. Furthermore, in mice, VEGF overexpression as an intervention to target the Hallmark of Aging “Altered intercellular communication” has been associated with improved health- and lifespan [28]. The PFA gene CCDC88A, which is associated with the molecular function “Vascular Endothelial Growth Factor Receptor Binding” is also associated with several other GO molecular functions, including the GOMF Epidermal Growth Factor. Secretion of the epidermal growth factor (EGF) and platelet-derived growth factor (PDGF) as part of the SASP can trigger activation and proliferation of progenitor cells [28], which further highlights the potential relevance of CCDC88A for age-related changes.

Increasing the number of mutual-information-ranked genes from our total pipeline for subsequent enrichment analyses can further increase the number of enrichment results, which might lead to further insights. However, as here and previously described [3], a relatively small number of PFA genes is already sufficient to distinguish between different groups or cell types validated in stage 8 in terms of information content to separate the clusters.

As a next step after the enrichment analysis, it is possible to analyze omics data and changes in protein expression to additionally check the *in silico* results [68]. This step, as well as the additional analysis of another suitable data set, are a cost- and time-efficient preliminary step before validating the results *in vitro* and *in vivo*. Nevertheless, when analyzing big data in biomedical research, the data quality and data integrity, as well as the comprehensiveness of the metadata, can hugely affect the resulting analyses, which needs to be considered when reusing a data set or publishing a data set [69].

### Future research (investigate the fine structure of clusters)

The pipeline described here can be further developed and applied to analyze the fine structure within clusters. Such a fine structure can originate in the fact that the gene expression data has been collected over time or during a cell transition from one type to another. Due to this process, a lot of intermediate steps could also be represented in the data set (not only the starting and final state of the cell transition). These cells in the intermediate state might have only minor differences from other cells. Consequently, the starting and the final cell states might not build separated clusters in an embedding, but rather both will be connected by the intermediate cell states. Such scenarios can be visualized by including the lifespan of a cell (experimental time) or if a data set is composed of several measurements of different time points after the starting time. Another option is to include the labels for the initial and final state cells (if experimentally determinable) or even some intermediate cell states (if known) from some annotation tools or other phenotypic characteristics known. Such labels can be included in the UMAP plot; see, e.g., https://umap-learn.readthedocs.io/en/latest/plotting.html for further details. If such labels/metrics are ordered within a cluster, it is a significant hint for some fine structure.

Once such a fine structure is present, a question similar to the one studied in this work is about which features/genes are of high predictive power to describe the fine structure within the cluster and, thus, the cell development. The presented pipeline is immediately ready to be applied in such use cases. After selecting only the single cells in the cluster with the fine structure of interest, one only has to define the correct output function where the label/metric information can be given. Such information can be the two-dimensional coordinates in the plane. This makes sense if, e.g., the initial states are located in one part of the cluster and the final states in a different part (e.g., opposite). Then, the first two rows in the combined data set in the find_cluster_differences are the output function (which then has two dimensions; set the parameter number_output_functions accordingly). This method will find corresponding important genes by which the coordinates can be predicted and thus might also contain the relevant information to model the transition from the initial to the final state.

Furthermore, the one-dimensional output setting (number_output_functions = 1) can be used when the fine structure in the cluster is given by a time label/metric (in case the time variable takes continuous values), like a lifespan or a running time of the experiment. In this case, the label data just needs to be replaced by time as an output function. While a label as the output function is discrete, time as the output function is continuous. However, our current implementation can deal with both kinds of output functions without any change. In the case of discrete intermediate steps, the framework can be used by just using the corresponding label representing the time order. The find_cluster_differences will then identify the genes that can predict the initial, intermediate, and final states, which models the characteristic genes for the transition.

In case the cluster describes a geometrical structure like a (bent) tube of the cells, in which one assumes fine structure or has some label or metric that shows the fine structure (e.g., initial state on one side and the final state on another side), one can fit a manifold to the cluster parametrized by one or two parameters. An easy example is a linear model x=(x1x2)=vt+c where *x*_1_, *x*_2_∈ℝ are the coordinates in the plane, v=(v1v2),c=(c1c2)∈R2 are parameters to be fitted, e.g., with a least-square method between the real coordinates of a data point y=(y1y2)∈R2 and its corresponding coordinates of the projection onto the manifold *x*_1_, *x*_2_. In this case, the manifold is the line with respect to the parameters *v* and *c*. The parameter *t*∈ℝ is consequently determined for each data point separately by the requirement to minimize the distance between the real coordinates of a data point *y* and *x* in the Euclidean distance. Consequently, for each data point to calculate *t*, which depends on the choice of *v* and *c*, we minimize the distance

$$\frac{1}{2}({\left(y_{1}-x_{1}(t)\right)}^{2}+{\left(y_{2}-x_{2}(t)\right)}^{2})=\frac{1}{2}({\left(y_{1}-v_{1}t-c_{1}\right)}^{2}+{\left(y_{2}-v_{2}t-c_{2}\right)}^{2})$$

which determines *t*. By deriving this expression with respect to *t*, setting the resulting gradient to 0 (characterization of the minimum of the distance function with respect to *t*), we obtain

$$t=\frac{(y_{1}-c_{1})v_{1}+(y_{2}-c_{2})v_{2}}{v_{1}^{2}+v_{2}^{2}}$$

which replaces *t* in the least-square fitting and thus making the parameter *t* a function of the optimization variables *v* and *c*. By such a procedure, the two-dimensional output function (providing the coordinates in two dimensions) is replaced by a one-dimensional output function (*t* as the coordinate within the manifold), and the parameter *t* of each data point replaces the corresponding label that was used in this present work. The find_cluster_differences can then be used to identify the genes that predict the parameter *t* for each cell, which means that we can describe the transition from the initial to the final state, which is encoded in the variable *t*.

A further option to transform a cluster into a manifold better fitting to its structure might be to turn the Euclidian coordinates into polar coordinates, where each data point has a distance from a midpoint and an angle. This representation might be beneficial if the (fine-) structure evolves from a midpoint since the genes are selected separately to describe the distance from the midpoint, modeling, e.g., steps of a development, and the angle, modeling, e.g., different cell fates.

## Conclusion

In this work, unlabeled feature selection determines objectively minimal gene sets separating groups and clusters. ML explainability methods transform the features correlating with phenotypic differences into causal reasoning, supported by additional pipeline and visualization tools, allowing user knowledge to further boost causal reasoning.

A pipeline for extracting objective characteristic features has been developed, and its application has been demonstrated with aging fibroblasts in the form of separating minimal gene sets. The pipeline consists of several techniques from data analytics and machine learning with the intention of identifying characteristic features for differences in phenotypic markers. For this purpose, a particular focus was put on visualization and ML explainability methods to check intermediate results with the pipeline and infuse domain knowledge about the biology involved in the data analysis. Human knowledge and artificial intelligence boost and complement each other in this way. It was shown that in terms of aging, genes that are relevant for important biological processes associated with the aging of humans and mice have been identified.

The whole strategy is fully documented and made available, thus opening up further strong application areas, e.g., regarding drug targets, applying the pipeline to heterogeneous tumor single-cell data to investigate differences between phenotypic markers. Examples are treated vs. untreated to study the mode of action or resistant vs. responding to find relevant genes making up the resistance. Since our ML method can distinguish different cell types without requiring further information, it is an uncomplicated and comfortable tool for analyzing differences between different cell types or different conditions, revealing genes that are important for distinguishing between the cell types or conditions. Due to the likely importance of the genes identified by this *in silico* analysis method, our ML approach might contribute to saving time and resources in both *in vitro* and *in vivo* research.

## Supporting information

S1 Data The file mutual_information0.csv provides the ranking of genes based on mutual information relevant to describe the differences between the two clusters with a purity of cells from young and old donors analyzed in this study. (ZIP)

S2 Data State of the GitHub repository of the characteristic feature extraction during submission period. (ZIP)

S3 Data State of the GitHub repository use for data preparation during submission period. (ZIP)
